# Morphological Computation Increases From Lower- to Higher-Level of Biological Motor Control Hierarchy

**DOI:** 10.3389/frobt.2020.511265

**Published:** 2020-10-21

**Authors:** Daniel F. B. Haeufle, Katrin Stollenmaier, Isabelle Heinrich, Syn Schmitt, Keyan Ghazi-Zahedi

**Affiliations:** ^1^Multi-Level Modeling in Motor Control and Rehabilitation Robotics, Hertie-Institute for Clinical Brain Research, University of Tübingen, Tübingen, Germany; ^2^Stuttgart Center for Simulation Science, Institute for Modelling and Simulation of Biomechanical Systems, University of Stuttgart, Stuttgart, Germany; ^3^Information Theory of Cognitive Systems, Max-Planck Institute for Mathematics in the Sciences, Leipzig, Germany

**Keywords:** morphological computation, control hierarchy, arm, motor control, muscles, preflexes

## Abstract

Voluntary movements, like point-to-point or oscillatory human arm movements, are generated by the interaction of several structures. High-level neuronal circuits in the brain are responsible for planning and initiating a movement. Spinal circuits incorporate proprioceptive feedback to compensate for deviations from the desired movement. Muscle biochemistry and contraction dynamics generate movement driving forces and provide an immediate physical response to external forces, like a low-level decentralized controller. A simple central neuronal command like “initiate a movement” then recruits all these biological structures and processes leading to complex behavior, e.g., generate a stable oscillatory movement in resonance with an external spring-mass system. It has been discussed that the spinal feedback circuits, the biochemical processes, and the biomechanical muscle dynamics contribute to the movement generation, and, thus, take over some parts of the movement generation and stabilization which would otherwise have to be performed by the high-level controller. This contribution is termed morphological computation and can be quantified with information entropy-based approaches. However, it is unknown whether morphological computation actually differs between these different hierarchical levels of the control system. To investigate this, we simulated point-to-point and oscillatory human arm movements with a neuro-musculoskeletal model. We then quantify morphological computation on the different hierarchy levels. The results show that morphological computation is highest for the most central (highest) level of the modeled control hierarchy, where the movement initiation and timing are encoded. Furthermore, they show that the lowest neuronal control layer, the muscle stimulation input, exploits the morphological computation of the biochemical and biophysical muscle characteristics to generate smooth dynamic movements. This study provides evidence that the system's design in the mechanical as well as in the neurological structure can take over important contributions to control, which would otherwise need to be performed by the higher control levels.

## 1. Introduction

In biological systems, voluntary movements are generated through a sequence of different processing units. From the motor cortex to the spinal cord to the stimulation signal running down the motor neuron to the muscle membrane. These processing units can be interpreted as a neurological, hierarchical control system (Loeb et al., 1999; Karniel, 2011). While it seems obvious that the neuronal structures are responsible for the initiation and execution of goal-directed movements, it has been discussed that also the morphology of a system contributes to the control (Iida and Pfeifer, 2004; Pfeifer and Iida, 2005; Paul, 2006; Blickhan et al., 2007; Ghazi-Zahedi et al., 2016). In particular in human arm movements, several control theories explicitly rely on the viscoelastic muscle characteristics to generate dynamic movements [e.g., impedance control Hogan, 1984, equilibrium point control Kistemaker et al., 2006, 2007a,b; Bayer et al., 2017]. Here, the muscles serve as a low-level zero-delay reflexes [termed *preflexes* Brown et al., 1995] capable of stabilizing the system against external perturbations (van Soest and Bobbert, 1993; Gerritsen et al., 1998; Loeb et al., 1999; Haeufle et al., 2010; Proctor and Holmes, 2010; John et al., 2013). Such contributions of the morphology have been termed “intelligence by mechanics” (Blickhan et al., 2007), “exploitive actuation” (Rieffel et al., 2010; Haeufle et al., 2012; Kalveram et al., 2012), or “morphological computation” (Pfeifer and Iida, 2005; Paul, 2006; Ghazi-Zahedi et al., 2016). Morphological computation, in this sense, captures the concept that control is partially performed by the controlled system interacting with the environment. More precisely, that part of the information processing necessary to generate a desired movement is performed by the morphological characteristics of the system, i.e., by its hard- or wet-ware.

Characterizing this contribution of the system's morphology to its behavior is possible by quantifying morphological computation (MC) (Zahedi and Ay, 2013; Ghazi-Zahedi et al., 2016; Ghazi-Zahedi, 2019). This requires a causal model of a reactive system's sensorimotor loop. The model must allow a clear separation of the system into a controller, actuator signals, sensor signals, and the physical system termed *world*, which includes the environment (in engineering this is typically called the *plant*). In a nutshell, the quantitative measure of morphological computation (MC_W_) then quantifies the contribution of the world state *W* and the actuator signal *A* to the further time evolution of the world state, i.e., the next world state *W*′. MC_W_ is high, if the current world state *W* has a strong influence on the next world state *W*′, i.e., the system exploits its physical properties. Thus, it is possible to quantify morphological computation in causal models where *A* and *W* can be observed, e.g., in neuro-muscular models (Ghazi-Zahedi et al., 2016).

The open question is, however, where in the biological control system *A* and *W* should be separated. Is *A* the output of the neurons that innervate the muscles (α motor neurons) and therefore initiate muscle contraction? Or is *A* much higher in the control hierarchy: the output of the central nervous system, i.e., the signals that initiate a movement? One could argue for the latter separation, as the decentralized low-level control circuits, like mono-synaptic reflexes, are hard-wired into the spinal cord and are therefore rather part of the system than part of the controller. Or has *A* even to be located much lower in the control hierarchy: the output force of the muscles? The argument for this level of separation would be that muscles with their non-linear viscoelastic properties serve as low-level zero-delay reflexes (*preflexes*) contributing to control. Furthermore, they adapt during our life-time to the requirements of our daily activities. From our point of view it is unclear where to separate between *W* and *A* and how this decision influences the calculation of MC. Furthermore, it is unclear, to which extend higher-level control can exploit morphological computation of the lower-level structures—in actual units of bit.

This is not only relevant for the understanding of biological systems, but also for bio-inspired and bio-mimetic robotics. Much effort has been taken to develop new robotic design concepts exploiting material properties (Kim et al., 2013; Rus and Tolley, 2015; Polygerinos et al., 2017), such as viscoelastic muscle-like actuators in arm movements (Boblan et al., 2004; Driess et al., 2018), elasticity in legged locomotion (Iida et al., 2009; Niiyama et al., 2012; Hutter et al., 2013; Sprowitz et al., 2013; Hubicki et al., 2016; Ruppert and Badri-Spröwitz, 2019) or morphology which empowers hopping (Nurzaman et al., 2015), goal-directed swimming (Manfredi et al., 2013), crawling Shepherd et al. (2011), or even grasping (Deimel and Brock, 2016). However, also in these approaches, the hierarchy of morphological computation has not yet been quantified.

The purpose of this study was therefore to investigate morphological computation in a hierarchical control system. The novelty of our approach was to quantify morphological computation on different control levels to better understand the hierarchy. This is relevant for two reasons: (1) it further evaluates and validates the quantification concept of MC and (2) shows how the biological control system may benefit from its hierarchical control structure and its non-linear actuators, i.e., the muscles. For this, we resort to computer simulations of human arm movements with a model that considers joint dynamics, muscles, reflexes, central pattern generators, and higher-level control.

## 2. Methods

To investigate morphological computation in a hierarchical control system, we simulate human arm movement with a neuro-musculoskeletal model (Stollenmaier et al., 2020a) (see also Supplementary Material). In this model, it is possible to access all state signals, i.e., the state of the control logic, the input to the low-level controller, the control signal, the muscles' active state (biochemistry), the muscles' force, the generated joint torques, and the resulting joint angles (section 2.1). Thus, we can access all levels of the neuro-muscular control hierarchy to quantify morphological computation (section 2.3).

### 2.1. Neuro-Muscular Model

The neuro-muscular model of human arm movements has been developed to study neuronal motor control concepts in the interaction with the musculoskeletal model. For this purpose, we combined a computational motor control model of goal-directed arm movements with a musculoskeletal model (Figure 2). We will shortly summarize the approach here and refer to the Supplementary Material for the details of the model.

**Figure 1:**
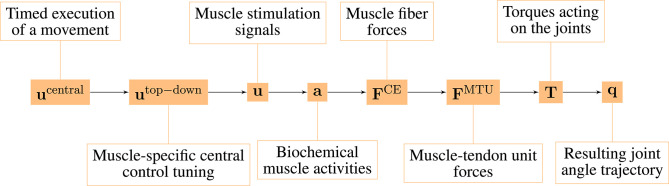
Overview over the hierarchy levels in our neuro-muscular model of the arm.

The model consists of several hierarchical layers (Figure 1), which we will describe shortly in the following, starting from the lowest hierarchical level (right-hand side). The chosen model parameters represent a generic man and are collected from different sources (van Soest and Bobbert, 1993; Kistemaker et al., 2006; Mörl et al., 2012; Bhanpuri et al., 2014 and others, listed in detail in the Supplementary Material).

**Figure 2:**
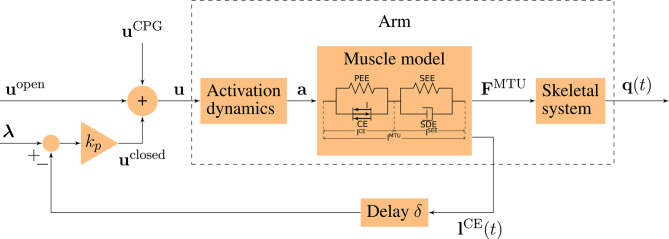
Schematic diagram of the motor control model. The motor command **u** is a sum of an open-loop and a closed-loop signal. The time-delayed feedback loop incorporates proprioceptive feedback (mono-synaptic reflexes) by comparing the actual muscle fiber lengths l^CE^(*t*) to desired values **λ**. **q**(*t*) = (φ(*t*), ψ(*t*)) contains the elbow and shoulder angle, respectively.

#### 2.1.1. Angles

**Figure 3:**
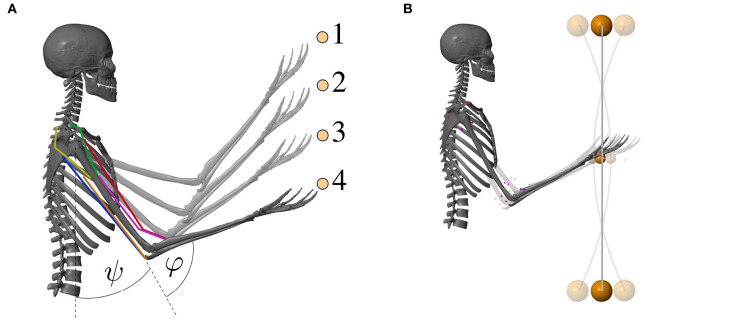
Visualization of the musculoskeletal model that was used for the computer simulations of the arm movements. The colored lines represent the modeled muscles. **(A)** Goal-directed point-to-point movement between the points 1–4 and **(B)** dynamic oscillation movements with a vibrating rod.

The musculoskeletal model predicts two-degree-of-freedom arm movements in the sagittal plane (see Figure 3). Its dynamics are determined by two rigid bodies (lower and upper arm) that are connected via two one-degree-of-freedom revolute joints that represent the shoulder and elbow joint. This can be described by double-pendulum equations of motion, i.e., second-order ordinary differential equations. The outputs of this layer are the predicted joint angles which correspond to the experimentally observable state (**q** ∈ ℝ^2^).

#### 2.1.2. Torques

The rigid bodies are driven by joint torques, which are calculated based on anatomical muscle paths (Hammer et al., 2019) and translating forces at the muscle origin, insertion, and via-points into joint torques. The outputs of this layer are the predicted joint torques (**T** ∈ ℝ^2^).

#### 2.1.3. Muscle-Tendon Unit Forces

Active forces are generated by six muscle-tendon units (MTUs), four monoarticular and two biarticular muscles. The force of each MTU is modeled using a Hill-type model accounting for muscle fiber and tendon characteristics (Haeufle et al., 2014). The dynamic of each MTU is modeled by a first-order ordinary differential equation. The outputs of this layer are the predicted muscle-tendon unit forces (**F**^MTU^ ∈ ℝ^6^).

#### 2.1.4. Muscle Fiber Forces

The model of the muscle fibers, termed contractile elements (CE), considers the dependence of the active fiber force on fiber length and contraction velocity known from biological muscle fibers. The outputs of this layer are the predicted muscle fiber forces (**F**^CE^ ∈ ℝ^6^).

#### 2.1.5. Biochemical Muscle Activity

The biochemical processes that lead from a neuronal muscle stimulation to a force generation can be modeled by a first-order ordinary differential equation. The implemented model of the activation dynamics further considers the fiber length dependency of this process (Hatze, 1977; Rockenfeller et al., 2015). The outputs of this layer are the predicted muscle fiber activity states (**a** ∈ ℝ^6^).

#### 2.1.6. Muscle Stimulation Signals

The bio-inspired hybrid equilibrium point controller exploits muscle characteristics by combining a feed-forward command [**u**^open^(*t*)] with spinal feedback on muscle fiber lengths [**u**^closed^(*t*)]. This feedback represents a simplified version of the mono-synaptic muscle spindle reflex, assuming that the muscle spindles provide accurate time-delayed information about the muscle fiber lengths **l**^CE^(*t*) (Kistemaker et al., 2006). The total motor command *u*_*i*_ for each muscle *i* is a sum of those components and is calculated as

(1) $$\begin{array}{l} \begin{array}{l} u_{i}\left(t\right)={\left\{u_{i}^{\text{open}}\left(t\right)+u_{i}^{\text{closed}}\left(t\right)+u_{i}^{\text{CPG}}\left(t\right)\right\}}_{0}^{1}\\ \;={\left\{u_{i}^{\text{open}}\left(t\right)+\frac{k_{p}}{l^{\text{CE,opt}}}\left(\lambda_{i}\left(t\right)-l_{i}^{\text{CE}}\left(t-\delta \right)\right)+u_{i}^{\text{CPG}}\left(t\right)\right\}}_{0}^{1}, \end{array} \end{array}$$

where *k*_*p*_ is a feedback gain and the time delay δ is set to 10 ms representing a short-latency reflex delay which is in a physiologically plausible range (More et al., 2010; Houk and Rymer, 2011). *l*^CE,opt^ stands for the optimal length of the contractile element. The operation ${\left\{x\right\}}_{0}^{1}$ sets values *x* < 0 to 0 and *x* > 1 to 1. The signal $u_{i}^{\text{CPG}}$ represents a central pattern generator (CPG). The outputs of this layer are the predicted muscle stimulation signals (α motor neuron activities **u** ∈ ℝ^6^).

#### 2.1.7. Muscle-Specific Central Control Tuning

The low-level controller gets two top-down input signals: The open-loop muscle stimulation $u_{i}^{\text{open}}\left(t\right)$ and the desired muscle fiber lengths λ_*i*_(*t*). Here, they represent an intermittent control approach, because they are piecewise constant functions over time. Herein, each constant value represents an *equilibrium posture* (EP), i.e., the system is in a stable equilibrium in these positions. The calculation of these central control signals for a given movement is described in detail in the Supplementary Material. The outputs of this layer are the top-down central commands to each low-level reflex circuit (**u**^top-down^ ∈ ℝ^12^).

#### 2.1.8. Timed Execution of a Movement

The output of our highest level of motor control is a single piecewise constant signal used to time the selection of equilibrium points meaning that all sub-circuits are switched at the same time (**u**^central^ ∈ ℝ^1^).

### 2.2. Simulation Experiments

#### 2.2.1. Movement 1: Point-to-Point Movements

The first movement investigated here is a point-to-point movement along a vertical line. Different movements between four target positions were evaluated (see Figure 3A and Supplementary Material). The central pattern generator is inactive for those movements [**u**^CPG^(*t*) = 0]. An animation of the movement is provided as Supplementary Material.

To consider the natural variation of this movement, we repeated the simulation of the movement 1 → 4 seven times. Each simulation only differed in the equilibrium postures (EPs) for the starting joint angles, the peak elbow joint angle, and the target joint angles. We determined these angles from motion capture data of a single subject performing the movement seven times. This natural variation of the angles resulted in different signals on the muscle-specific central control level, i.e., different **u**^top-down^ signals. All other parameters of the controller were kept constant.

#### 2.2.2. Movement 2: Dynamic Oscillatory Movements

For the second movement, a vibrating rod was added to the hand in the model (see Figure 3B). The technical specifications of the rod can be found in the Supplementary Material. To excite the rod, as done in training and rehabilitation exercises, a sinusoidal signal **u**^CPG^ mimicking the output of a central pattern generator (CPG) is added to the motor command **u**:

(2) $$\begin{array}{l} {\text{u}}^{\text{CPG}}\left(t\right)=\hat{u}\cdot \sin\left(2\pi \cdot f^{\text{CPG}}\cdot t+\phi_{0}\right), \end{array}$$

with *û* = 0.1: amplitude, *f*^CPG^: frequency, ϕ_0_: phase. The muscles are synchronized by setting ϕ_0_ = 0 for flexing muscles and ϕ_0_ = π for extending muscles.

The oscillation is exited for 0 ≤ *t* ≤ 4*s*. After this, **u**^CPG^ = 0 and the oscillation is then only a result of the dynamics of the system and not of the controller anymore. An animation of the movement is provided as Supplementary Material.

To consider the natural variation of this movement, we analyzed the frequency pattern of a single subject performing a swing-rod exercise. The fast-fourier-transform spectrum indicates a frequency variance of 0.2*Hz*. We therefore repeated the simulation 14 times with a set of random CPG frequencies *f*^CPG^ = 3.8 ± 0.2*Hz*.

#### 2.2.3. Details on the Human Experiments to Estimate Natural Variability

Two healthy subjects participated in the study. The experimental procedure was approved by the local ethics committee (886/2018BO2). All participants gave their informed consent prior to participation. The movements were recorded with a 12-camera motion capturing system (Vicon Motion Systems Ltd, UK) using a marker set with 29 retro-reflecting markers. Using the recorded marker positions over time, shoulder and elbow angles were reconstructed (Rettig et al., 2009). The reconstructed joint angle trajectories were smoothed with a Savitzky-Golay polynomial filter (of order 4 and with a window size of 41 sampling points).

### 2.3. Quantifying Morphological Computation

The following paragraphs will only give a brief introduction to the quantification of MC. For a full discussion on this issue, please read (Ghazi-Zahedi, 2019) or the Supplementary Material to this publication. Quantifying MC requires a causal model of the sensorimotor loop which divides a cognitive system into a brain, actuators, environment, and sensors. In the context of this work, we are focusing on reactive systems which means that the actuators are directly connected with the sensors. A cognitive system is then fully described by the following set of Markov processes:

(3) $$\begin{array}{l} \beta :W\to \Delta_{S}\;[\beta \left(s|w\right)] \end{array}$$

(4) $$\begin{array}{l} \pi :S\to \Delta_{A}\;[\pi \left(a|s\right)] \end{array}$$

(5) $$\begin{array}{l} \alpha :W\times A\to \Delta_{W}\;[\alpha \left(w^{\prime }|w,a\right)], \end{array}$$

where w∈W is the value of the world state *W*, s∈S is the value of the sensor state *S*, and a∈A is value of the actuator state *A*. We call β(*s*|*w*) the sensor map, as it describes how the agents perceive the environment, π(*a*|*s*) the policy, as it describes how the agent chooses an action as a reaction to a sensor, and finally we call α(*w*′|*w, a*) the world dynamics kernel, as it describes how the next world state *W*′ depends on the current world state *W* and the current action *A*. It is important to note here that the world state *W* captures everything physical. This means that the world state *W* captures the state of the system's body and its environment.

To quantify MC, we take a closer look at the world dynamics kernel α(*w*′|*w, a*). Assume that the next world state *W*′ does not depend on the current world state *W* but only on the current action *A*. This means that the world dynamics kernel reduces to $\tilde{\alpha }\left(w^{\prime }|a\right)$. In this case, it is fair to say that the system shows no MC at all, since the behavior is fully controlled by the action *A*. Any measured divergence from this assumption means that the current world state *W* had an influence on the next world state *W*′, and hence, the system is exploiting the physical properties of its body and its interactions with the environment. This can be measured by the Kullback-Leibler Divergence (Cover and Thomas, 2006) in the following way:

(6) $$\begin{array}{l} {\text{MC}}_{\text{W}}:=\underset{w^{\prime },w,a}{\sum }p\left(w^{\prime },w,a\right)\log_{2}\frac{\alpha \left(w^{\prime }|w,a\right)}{\tilde{\alpha }\left(w^{\prime }|a\right)}. \end{array}$$

The output of our models contains discrete numerical data, i.e., *S*, *A*, and *W* are discrete variables. Therefore, we will summarize the approach for discrete variables here. For a discussion on how to estimate MC_W_ on continuous state spaces, please see Ghazi-Zahedi (2019).

The joint distribution *p*(*w*′, *w, a*) can be estimated by a frequency method, i.e., by counting the number of occurrences of each triplet (*w*′, *w, a*) normalized by the number of samples in the data. This leads to the following estimation for *p*(*w*′, *w, a*):

(7) $$\begin{array}{l} p\left(w^{\prime },w,a\right)=\frac{c_{w^{\prime },w,a}}{N}, \end{array}$$

where $c_{w^{\prime },w,a}$ is the number of occurrences of (*w*′, *w, a*) and *N* is the total number of samples.

MC_W_ can now be calculated in the following way:

The value calculated in line 9, MC_W_, represents the morphological computation primarily used in this work. Sometimes it is further interesting to take a look at the state-dependent morphological computation, i.e., the time evolution of the quantity. This requires minimal changes to the original algorithms. Instead of calculating the probability-weighted sum over all states (line 9 in Algorithm 1), which leads to a single number as a result, the measures are evaluated *n*-tuple in the data set. This means that for MC_W_, the logarithm is evaluated for every triple *w*_*t*+1_, *w*_*t*_, *a*_*t*_ (see Algorithm 2).

**Algorithm 1:** Algorithm for MC_W_.

1: $p\left(w^{\prime },w,a\right)\leftarrow {\left(0\right)}_{\left|W|\times |W|\times |A\right|}$ {Matrix with |*W*| × |*W*| × |*A*| entries set to zero}
2: **for** *t* = 1, 2, …, *T* − 1 and $w_{t+1},w_{t}\in w^{*},a_{t}\in a^{*}$ **do**
3: *p*(*w*_*t*+1_, *w*_*t*_, *a*_*t*_) ← *p*(*w*_*t*+1_, *w*_*t*_, *a*_*t*_) + 1
4: **end for**
5: *p*(*w*′, *w, a*)←*p*(*w*′, *w, a*)/(*T* − 1)
6: Estimate *p*(*w*′, *a*) from w^*^, a^*^ or by summing over *w*
7: $p\left(w^{\prime }|w,a\right)=p\left(w^{\prime },w,a\right)/\underset{w^{\prime }}{\sum }p\left(w^{\prime },w,a\right)$
8: $p\left(w^{\prime }|a\right)=p\left(w^{\prime },a\right)/\underset{w^{\prime }}{\sum }p\left(w^{\prime },a\right)$
9: ${\text{MC}}_{\text{W}}=\underset{w^{\prime },w,a}{\sum }p\left(w^{\prime },w,a\right)\log_{2}\frac{p\left(w^{\prime }|w,a\right)}{p\left(w^{\prime }|a\right)}$

**Algorithm 2:** Algorithm for state-dependent MC_W_(*t*).

1: Perform steps 1–8 from Alg. 1
2: **for** *t* = 1, 2, …, *T* − 1 and *w*′, *w* ∈ w^*^, *a* ∈ a^*^ **do**
3: ${\text{MC}}_{\text{W}}\left(t\right)=\log_{2}\frac{p\left(w^{\prime }|w,a\right)}{p\left(w^{\prime }|a\right)}$
4: **end for**

In conclusion, in order to quantify MC, we need time signals of the World and Actuator states, *W* and *A*, respectively. This means that it is necessary to separate the state variables of the system into *W* and *A*.

The neuro-muscular model investigated here has several hierarchical levels (Figure 1). For this study, we systematically separated the state variables between all of these different hierarchy levels and calculated MC for each possible hierarchy level.

**Figure 4:**
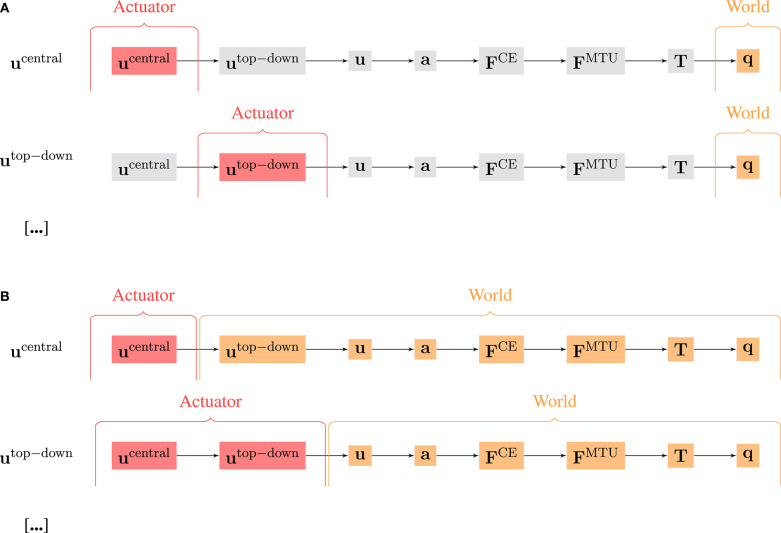
Visualization of the difference of the calculation of MC_W_ using **(A)** selected and **(B)** accumulated hierarchy levels as actuator signal A and world state W. Note that for the oscillation movements, the observable state **q** includes both the joint angles and the rod position.

There are two possible approaches to select *W* and *A* and then calculate MC (Figure 4): The first approach (Figure 4A) relates to the evaluation of experimental data, where usually not all state variables can be recorded (especially in biological systems). Here, *W* is always the mechanical system state **q**(*t*), i.e., the joint positions (and for the oscillation movement also the position of the rod mass relative to the hand). *A* on the other hand contains only signals of one hierarchy level. We term this approach “selected hierarchy levels” and term the respective morphological computation ${\text{MC}}_{\text{W}}^{\text{sel}}$.

The second approach (Figure 4B) always includes all signals. It represents a clear cut at a specific level. All signals below this cut-level are combined into *W* and all above into *A*. We termed this approach “accumulated hierarchy levels” and termed the respective morphological computation ${\text{MC}}_{\text{W}}^{\text{acc}}$.

### 2.4. Statistical Analysis

Each simulation run provides data to calculate morphological computation on all different hierarchy levels. Each hierarchy level is then quantified by a single scalar quantity MC_W_ representing the respective morphological computation (see line 9 in Algorithm 1). By varying the control parameters as described above, the resulting MC_W_ values represent a natural variation for the same movement. The hypothesis (H_0_) was that there is no significant difference in MC between hierarchy levels across all repetitions of the movement. Each hierarchy thus represents a different group and we used ANOVA to test whether these groups differ. The normal distribution was tested with a Shapiro-Wilk test (with α = 0.1 to keep the beta error in check). The test confirmed normal distribution in the majority of the groups (17 out of 28). This should not influence the result, since ANOVA is robust to deviations form normal distribution, especially here where each group has the same number of samples. As the different hierarchy levels are taken from the same simulation, they are not independent. To test their statistical difference, we therefore analyzed the data with a repeated measures ANOVA. We further used a pairwise *post-hoc* test with Bonferroni correction to analyze which levels actually differ.

## 3. Results

**Figure 5:**
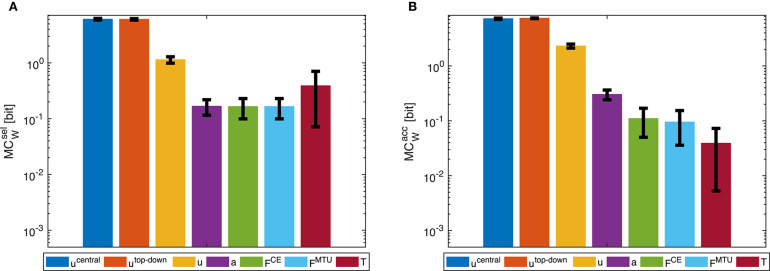
Point-to-point movement: Morphological computation MC_W_ on different hierarchy levels for an exemplary point-to-point movement (1 → 4, see Figure 3A). Morphological computation was evaluated using **(A)** selected (${\text{MC}}_{\text{W}}^{\text{sel}}$) and **(B)** accumulated hierarchy levels (${\text{MC}}_{\text{W}}^{\text{acc}}$). Note that a logarithmic scale is used for the y-axis. Shown are the mean ±1.96 times standard deviation (≈95% confidence interval) of seven simulation runs with different starting, intermittent, and target equilibrium postures taken from the natural variation observed in a human experiment. As tested by an ANOVA, there are significant differences in MC_W_ between the different hierarchy levels. The pairwise *post-hoc* test revealed that for ${\text{MC}}_{\text{W}}^{\text{sel}}$ there are two groups with similar mean: the highest two levels *u*^central^, *u*^top-down^, and the three levels *a*, *F*^CE^, and *F*^MTU^. The levels *u* and *T* differ from all others. For ${\text{MC}}_{\text{W}}^{\text{acc}}$, the three lowest levels *F*^CE^, *F*^MTU^, and *T* are one group. All other levels differ from all others. All significance levels were set to *p* < 0.05. The limit of the y-axis is set to the maximum MC value that would result from having a constant signal as input. Plots of the results of the other movements can be found in the Supplementary Material, but show the same trends.

Morphological computation is highest for the most central level of the control hierarchy investigated here (**u**^central^). This holds for all four types of point-to-point movements we evaluated (Figure 5) as well as for the dynamic oscillation movement (Figure 7). Going further down in the control hierarchy, MC always decreases for the accumulated scenario (${\text{MC}}_{\text{W}}^{\text{acc}}$), and almost always for the selected (${\text{MC}}_{\text{W}}^{\text{sel}}$) with one exception: the torque. Choosing the torque **T** as actuator signal, the value for MC is higher than using one of the next higher-level signals of actuation. Please note that the figures are shown in logarithmic scale to allow a better comparison of the large differences between MC for the different hierarchy levels.

In general, using accumulated hierarchy levels results in smaller morphological computation than using selected hierarchy levels (${\text{MC}}_{\text{W}}^{\text{acc}}<{\text{MC}}_{\text{W}}^{\text{sel}}$). Furthermore, pointing movements have a lower morphological computation than the dynamic oscillation movements.

The reproduction of the experimentally observed variation of the movement 1 → 4 in simulation also leads to a variation of MC_W_. This variation is relatively small compared to the overall difference between hierarchy levels. Therefore, an ANOVA test reveals statistical significant differences between the hierarchy levels. However, not all levels are significantly different. Especially *u*^central^ and *u*^top-down^, as well as *F*^CE^ and *F*^MTU^ do not differ significantly in MC.

### 3.1. Noise in Point-to-Point Movements

In the pointing movements, all state variables are smooth, which is a result of the noise-free formulation of the continuous control signals. Therefore, the highest control levels produce very simple control signals, i.e., piecewise constant signals in time (see above and Supplementary Material for more details).

**Figure 6:**
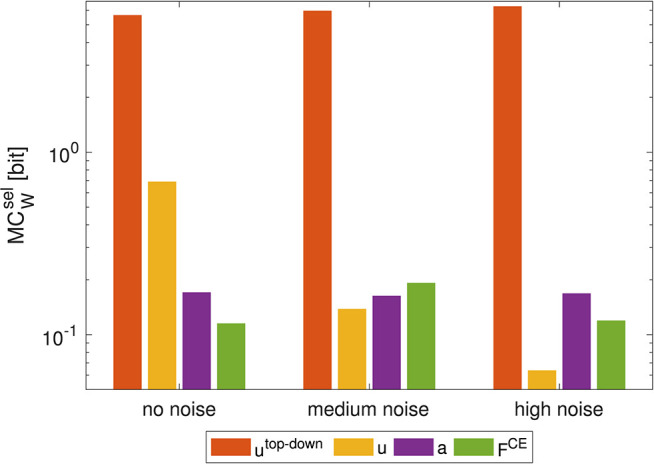
Influence of noise on morphological computation. Morphological computation for selected hierarchy levels (${\text{MC}}_{\text{W}}^{\text{sel}}$) for a point-to-point movement (1 → 4). The noise was added to the muscle stimulation **u** [noise levels: medium: 40/300 · (*u*_max_ − *u*_min_), high: 80/300 · (*u*_max_ − *u*_min_)]. As a result, ${\text{MC}}_{\text{W}}^{\text{sel}}$ at the muscle stimulation level decreases and increases in adjacent hierarchy levels. Note that a logarithmic scale is used for the y-axis.

To test whether this smooth definition has an influence on the result, we added random (uniformly distributed) noise to the muscle stimulation signals **u** [noise levels: medium: 40/300 · (*u*_max_ − *u*_min_), high: 80/300 · (*u*_max_ − *u*_min_)]. This changes the previously consistent trend: the higher the added noise, the lower the MC at the level of the muscle stimulation **u** (Figure 6). At the same time, MC between at the muscle activity level **a** increases. This leads to the fact, that—after adding noise to the stimulation signal - MC with **u** as actuator signal is lower than the calculation with **a** as actuator signal. However, this change in trend is only true if morphological computation is evaluated on selected signals (${\text{MC}}_{\text{W}}^{\text{sel}}$). For ${\text{MC}}_{\text{W}}^{\text{acc}}$, the trend is never reversed. Noise only slightly shifts the values (not shown).

### 3.2. Dynamic Oscillatory Movements

**Figure 7:**
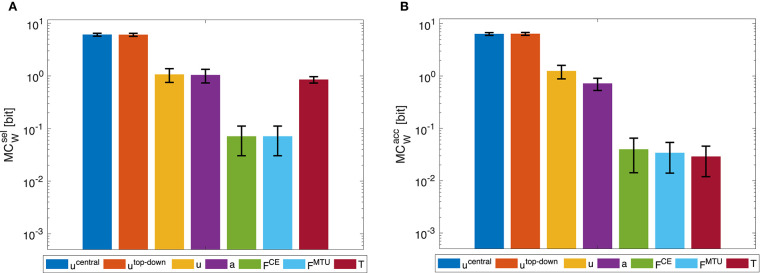
Dynamic oscillation movement: Morphological computation MC_W_ for **(A)** selected (${\text{MC}}_{\text{W}}^{\text{sel}}$) and **(B)** accumulated hierarchy levels (${\text{MC}}_{\text{W}}^{\text{acc}}$). Shown are the mean ±1.96 times standard deviation (≈95% confidence interval) of 14 simulation runs with a set of random CPG frequencies in the spectrum observed in a human experiment. As tested by an ANOVA, there are significant differences in MC_W_ between the different hierarchy levels. The pairwise *post-hoc* test revealed that for ${\text{MC}}_{\text{W}}^{\text{sel}}$, the highest levels *u*^central^ and *u*^top-down^ have similar means, so do the muscle stimulation *u*, activity *a*, as well as the forces *F*^CE^ and *F*^MTU^. Only the torque level *T* differs from all other groups. For ${\text{MC}}_{\text{W}}^{\text{acc}}$, the highest levels *u*^central^ and *u*^top-down^ have similar means, so do the lowest levels *F*^CE^, *F*^MTU^, and *T*. All significance levels were set to *p* < 0.05. The limit of the y-axis is set to the maximum MC value that would result from having a constant signal as input. Note that a logarithmic scale is used for the y-axis.

The general trend of decreasing morphological computation for lower hierarchy levels was the same in the dynamic oscillation movements (Figure 7).

**Figure 8:**
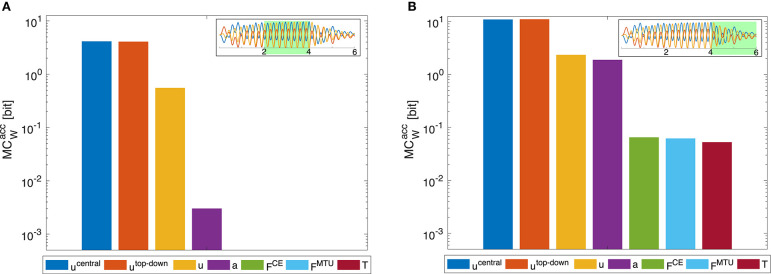
Dynamic oscillation movement. Morphological computation is higher for the last movement phase where the central pattern stimulation is deactivated and the movement continues due to the passive dynamics of the arm-rod system. **(A)** ${\text{MC}}_{\text{W}}^{\text{acc}}$ evaluated for the time span between 2 and 4 s **(B)** for the time span between 4 and 6 s, as indicated by the insets, which show the oscillation of the joints and the rod (cf. Figure 9). In the first time span, the control signals are sinusoidal muscle stimulations exciting the rod at its resonance frequency (*f*^CPG^ = 3.8*Hz*). In the second time span **(B)**, the sinusoidal stimulation is zero and the oscillation is only driven by the dynamics of the rod. The limit of the y-axis is set to the maximum MC value that would result from having a constant signal as input. Note that a logarithmic scale is used for the y-axis.

**Figure 9:**
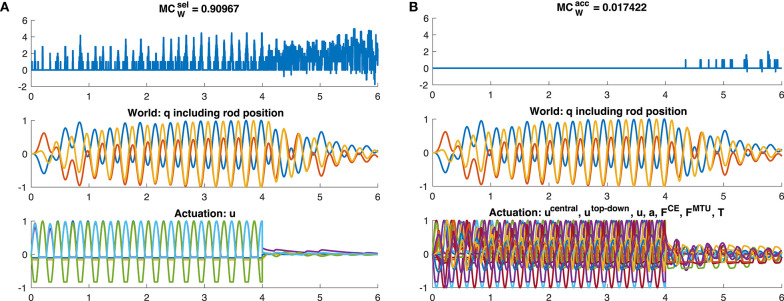
Time evolution of morphological computation MC_W_, world state *W* and actuator state *A* for the dynamic oscillation movement. The oscillation is exited for 0 ≤ *t* ≤ 4*s* by a sinusoidal CPG stimulation signal. After this, *u*^CPG^ = 0 and the oscillation is then only a result of the dynamics of the system and not of the controller anymore. Shown here is exemplary **(A)** the case of ${\text{MC}}_{\text{W}}^{\text{sel}}$ of the muscle stimulation level *u* (yellow bar in Figure 7A) and **(B)** the case of ${\text{MC}}_{\text{W}}^{\text{acc}}$ including only the joint angles and rod position as world state (dark red bar in Figure 7B).

However, the dynamic oscillation data has different phases. In the initial phase (*t* ≤ 4*s*), the rod is excited by sinusoidal muscle stimulation signals with a frequency tuned to the rod's resonance (Figure 9). In this phase, everything oscillates in sync and the morphological computation is on average smaller. Once the CPG is turned off (*t* > 4*s*), the control signals become relatively steady—only influenced by the feedback signals trying to hold the position. The rod, however, still has a lot of energy and therefore keeps oscillating. In this phase, MC_W_ increases. These results are similar on all levels of the control hierarchy (Figure 8). Interestingly, ${\text{MC}}_{\text{W}}^{\text{acc}}$ actually becomes zero on the lower hierarchy levels in the resonance oscillating movements between 2 ≤ *t* ≤ 4*s*. This means that muscle fiber force **F**^CE^, muscle-tendon unit force **F**^MTU^, and joint torques **T** contain the same information as the mechanical state of the system **q**.

## 4. Discussion

The meaning of morphological computation can be seen quite well in the example of the dynamic oscillations. In the initial phase, the controller enforces a dynamic oscillation at the system's resonance. In resonance, the morphological computation is then quite low, as most—or even all—information on the system state is already contained in the stimulation, activity, and muscle force signals (Figures 8A, 9). This is similar to a robotic model, driven by complex control signals (Ghazi-Zahedi et al., 2016). However, if the sinusoidal excitation is switched off, the rod dynamics take over and generate a rich dynamic behavior at almost no information input on the control/actuation levels. Hence, morphological computation is high (Figure 8B). This case is similar to, e.g., mechanical toys, such as passive dynamic walkers which generate the entire behavior based on their mechanical properties. This example confirms that the measure of MC_W_ captures what we would expect as morphological computation.

By measuring morphological computation in a hierarchical control system, we can—for the first time—quantify the contribution of different hierarchy-levels to the control. The increase of morphological computation for higher-levels of the control hierarchy in the accumulative evaluation (${\text{MC}}_{\text{W}}^{\text{acc}}$) means that the lower control levels actually contribute quite significantly. To be able to test whether the differences between the hierarchy-levels are significant, we introduced variations based on experimental data. Not all MC data generated in this way fulfills the ANOVA assumption of equal distribution for each group represented by a hierarchy level. Still, the results found by the ANOVA and *post-hoc* test match what can be seen in Figures 5, 7. Literature suggests this contribution of muscles to dynamic movements (van Soest and Bobbert, 1993; Gerritsen et al., 1998; Wagner and Blickhan, 1999; Eriten and Dankowicz, 2009; van der Krogt et al., 2009; Haeufle et al., 2010, 2012, 2020; Pinter et al., 2012; John et al., 2013; Kambara et al., 2013; Bayer et al., 2017; Stollenmaier et al., 2020a). In this sense, the ${\text{MC}}_{\text{W}}^{\text{acc}}$ quantifies the “importance” of each hierarchical level in the sense of influence on the behavior (world state evolution) of the system. This approach shows that the muscle-driven arm movements can be initiated with very little information on the top control levels while the lower control levels and also the biochemical and muscular dynamics generate a smooth information-rich signal and ultimately dynamic behavior out of these reduced signals (Kistemaker et al., 2006; Stollenmaier et al., 2020a). This is reflected in the large differences in ${\text{MC}}_{\text{W}}^{\text{acc}}$ (please note that the plots use a logarithmic scale).

We expect similar results for robotic arm systems that employ muscle-like actuation, e.g., fluidic muscles (Boblan et al., 2004; Driess et al., 2018). Fluidic muscles show muscle-like force-length-velocity characteristics (Klute et al., 2002) and by antagonistic co-contraction allow for variable joint stiffness (Wolfen et al., 2018). This way, even simple piecewise constant control signals will result in smooth dynamic movements (Driess et al., 2018), very similar to what is known from simulation results (Kistemaker et al., 2007a; Stollenmaier et al., 2020a,b; Wochner et al., 2020), and are hypothesized to be a control principle of goal-directed arm movements (Feldman and Levin, 2009). Furthermore, as mechanical (visco-)elastic morphological characteristics are also known to benefit robotic locomotion (Iida et al., 2009; Shepherd et al., 2011; Niiyama et al., 2012; Hutter et al., 2013; Manfredi et al., 2013; Sprowitz et al., 2013; Nurzaman et al., 2015; Hubicki et al., 2016; Ruppert and Badri-Spröwitz, 2019), we expect that such a hierarchy in morphological control may be present in such systems too. This will become especially interesting if hierarchical control systems learn to exploit these morphological contributions to efficiently generate movements (e.g., Manoonpong et al., 2007; Driess et al., 2018; Büchler et al., 2020).

### 4.1. Difference Between the Two Approaches to Calculate MC

The ${\text{MC}}_{\text{W}}^{\text{acc}}$ approach is particularly of value for the evaluation of hierarchical computational models of motor control, where all system states are observable. The calculation of morphological computation only based on selected actuation signals ${\text{MC}}_{\text{W}}^{\text{sel}}$, however, better represent the experimenters' reality, where most of the system states are not or hardly observable. While the general trend is the same, we observed that for the joint torques the morphological computation increases again. This can be attributed to the fact that the two joint torque signals contain less information than the six muscle force signals. Furthermore, ${\text{MC}}_{\text{W}}^{\text{sel}}$ is influenced by noise. Increasing noise increases the apparent information content of the signals and thus reduces morphological ${\text{MC}}_{\text{W}}^{\text{sel}}$ (yellow bar in Figure 6). Interestingly, this additional noise is basically filtered by the low-pass filter characteristics of the muscles' activation and contraction dynamics resulting in quite similar output behavior. Therefore, ${\text{MC}}_{\text{W}}^{\text{sel}}$ increases for the lower hierarchy levels. The consequence of this is, that one has to be careful if applying ${\text{MC}}_{\text{W}}^{\text{sel}}$ to experimental data, as noise on the signals may alter the result.

### 4.2. Model Considerations

The model used in this study was chosen as it resembles the coarse organ-level dynamics of the neuro-musculoskeletal system that leads to goal-directed movements. However, it does not consider that in reality, each muscle-tendon unit consists of many motor units that have to be and can be controlled separately by higher control levels. We cannot rule out that these principles of the biological system will have a significant effect on the overall morphological computation and its distribution among the hierarchy levels. In principle, this could be investigated in more detailed models (e.g., Heidlauf and Röhrle, 2013; Mordhorst et al., 2015). However, our model represents the basic functional unit (Schmitt et al., 2019) considering the main dynamic properties relevant for the passive contribution of muscles to control (Pinter et al., 2012). Furthermore, the two movements investigated here represent primitives that could potentially be combined to generate more complex arm movements (Sternad et al., 2000; Wei et al., 2003). Therefore, we expect that our findings represent a fundamental concept in biology. We further expect that it extends to other movements too, e.g., locomotion, for which it is known that muscles significantly contribute to the movement generation (van Soest and Bobbert, 1993; Gerritsen et al., 1998; Daley et al., 2009; Haeufle et al., 2010; John et al., 2013) and allow to simplify higher-level control (Haeufle et al., 2014, 2020; Ghazi-Zahedi et al., 2016).

Overall, we here provide evidence that the systems' design in the mechanical as well neurological structure facilitates the control task by providing an appropriate integration of signals at different levels of the control hierarchy.

## Data Availability Statement

The datasets generated for this study are available on request to the corresponding author. The code to calculate morphological computation is online available: https://github.com/kzahedi/gomi.

## Author Contributions

DH and KG-Z: study design. DH, KS, IH, and SS: modeling and simulation. DH and KS: data evaluation. DH, KS, IH, SS, and KG-Z: manuscript. All authors contributed to the article and approved the submitted version.

## Conflict of Interest

The authors declare that this study received funding from the company Haider Bioswing. The funder provided us with two vibrating rods free of charge, but no monetary funding. The funder was not involved in the study design, collection, analysis, interpretation of data, the writing of this article or the decision to submit it for publication.

## Figures and Tables

**Table:** 

**u**^central^ ∈ ℝ	timed execution of the movement (EP timing)
**u**^top-down^ ∈ ℝ^12^	muscle-specific central control tuning (*u*^*open*^ and λ)
**u** ∈ ℝ^6^	muscle stimulation signals
**a** ∈ ℝ^6^	biochemical muscle activities
**F**^CE^ ∈ ℝ^6^	muscle fiber forces [N]
**F**^MTU^ ∈ ℝ^6^	muscle-tendon unit forces [N]
**T** ∈ ℝ^2^	torques acting on the joints [Nm]
**q** = (φ, ψ) ∈ ℝ^2^	joint angle trajectory
**λ** ∈ ℝ^6^	desired muscle fiber lengths [m]
**l**^CE^ ∈ ℝ^6^	muscle fiber length [m]
*l*^CE, opt^ ∈ ℝ	optimal *l*^CE^ length [m]
*k*_*p*_ ∈ ℝ	feedback gain
δ ∈ ℝ	feedback delay [s]
**u**^open^ ∈ ℝ^6^	open-loop control signal
**u**^closed^ ∈ ℝ^6^	closed-loop control signal
**u**^CPG^ ∈ ℝ^6^	central pattern generator (CPG) signal
û ∈ ℝ	amplitude of the CPG
*f* ∈ ℝ	frequency of the CPG
ϕ_0_ ∈ ℝ	phase of the CPG

**Table:** 

MC_W_	quantitative measure of morphological computation
${\text{MC}}_{\text{W}}^{\text{sel}}$	*MC*_*W*_ for selected hierarchy levels
${\text{MC}}_{\text{W}}^{\text{acc}}$	*MC*_*W*_ for accumulated hierarchy levels
*W*	current world state
*W*^′^	next world state
*A*	actuator state
*S*	sensor state
w∈W	value of *W*
a∈A	value of *A*
s∈S	value of *S*
α(*w*^′^|*w, a*)	world dynamics kernel
β(*s*|*w*)	sensor map
π(*a*|*s*)	policy
*p*	probability distribution
$c_{w^{\prime },w,a}$	number of occurrences of (*w*^′^, *w, a*)
*N*	total number of samples

## Glossary

### List of Symbols

Model levels and control variables.

Morphological computation.
