# Supplementary material for: Morphological Computation Increases From Lower- to Higher-Level of Biological Motor Control Hierarchy
Source: Front Robot AI. 2020 Oct 21;7:511265. doi: 10.3389/frobt.2020.511265 (PMC7805613; doi:10.3389/frobt.2020.511265)
Supplement: Supplementary file 1 [file Data_Sheet_1.pdf]

Supplementary material to "Morphological computation increases from lower- to higher-level of biological motor control hierarchy"

Daniel F B Haeufle[1], Katrin Stollenmaier[1], Isabelle Heinrich[1]
Syn Schmitt[2], and Keyan Ghazi-Zahedi[3]

[1]Multi-Level Modeling in Motor Control and Rehabilitation Robotics,
Hertie-Institute for Clinical Brain Research, University of Tübingen, Germany.

[2]Institute for Modelling and Simulation of Biomechanical Systems and
Stuttgart Center for Simulation Science, University of Stuttgart, Germany.

[3]Max-Planck Institute for Mathematics in the Sciences, Leipzig, Germany.

Contents

1 Description of the human experiments **2**
- 1.1 Experimental setup: general description . . . 2
- 1.2 Experimental setup: Point-to-point movements . . . 3
- 1.3 Experimental setup: Oscillation movements . . . 3
- 1.4 Experimental results: Point-to-point movements . . . 3
- 1.5 Experimental results: Oscillation movements . . . 4

2 Supplementary simulation results point-to-point movements **5**

3 More details on quantifying morphological computation **7**
- 3.1 Causal Model of the Sensorimotor Loop . . . 7
- 3.2 Quantifying Morphological Computation . . . 8
- 3.3 Estimating Quantifications of Morphological Computation . . . 9

4 The arm model *Arm26* **10**
- 4.1 Musculoskeletal model of the arm: Mechanics and Actuation . . . 10
- 4.2 Equilibrium Point Controller . . . 17
  - 4.2.1 Optimization of the control parameters for a point-to-point movement . . . 17
- 4.3 Dynamic oscillation movements with vibrating rod . . . 18

1 Description of the human experiments

The sole purpose of the experiments was to get a quantitative basis for the variations of the simulated movements. These variations allowed us to statistically test the main hypothesis of this paper: that there is a difference in morphological computation between different hierarchy levels.

1.1 Experimental setup: general description

A quantitative movement analysis by a VICON motion capture system with 12 cameras was used to quantify the participants' movements. The system captures three-dimensional movement trajectories of the subjects. The spatial resolution of the system was approximately 1 mm. All trials were recorded at a sampling rate of 120 Hz, using 29 retro-reflecting markers that were positioned based on the Heidelberger Upper Extremity (HUX) model (Rettig et al., 2009).

The HUX model consists of seven segments and determines the individual's joint center of the shoulder and elbow joint with one static and three dynamic calibrations per subject. The calibration movements include shoulder abduction and adduction, shoulder anteversion and retroversion (both performed with zero elbow flexion) and elbow flexion and extension, all with the participant's maximal range of motion. Additional to the HUX marker set, further markers were added to evaluate the finger position (point-to-point movements) and to the swing rod (oscillating movements). All markers are listed in Table 1.

Marker	Anatomical Position
**HUX model:**	
HEAD	central forhead
R/L MAS	above mastoids (right/left)
CLAV	jugular notch
C7	thoracic vertebrae cervicalis 7
T10	thoracic vertebrae thoracalis 10
SACR	mid-way between posteriorsuperior iliac spines
R/L ASI	anterior superior iliac spines (right/left)
R/L SHO	acromio-clavicular joint (right/left)
R/L HUMS	tuberositas deltoidea (right/left)
R/L ELB	twin-marker ulna 2 cm distally to the olecranon (right/left)
R/L ELBW	twin-marker ulna 2 cm distally to the olecranon (right/left)
R/L ULN	processus styloideus ulnae (right/left)
R/L RAD	processus styloideus radii (right/left)
**additional markers:**	
R 1PD	1st distal interphalangeal
R 2MCP	2nd articulationes metacarpophalangeae
R 2PD	2nd phalanx distalis
R 5MCP	5th articulationes metacarpophalangeae

Table 1: Marker placement according to the HUX model with additional markers to determine the finger position.

1.2 Experimental setup: Point-to-point movements

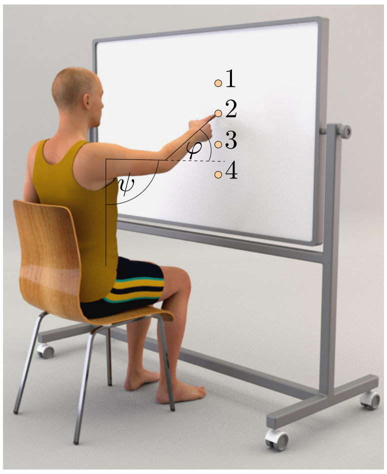

Figure 1: Sketch of the experimental setup. Using a motion capture system, elbow and shoulder angles of fast goal-directed pointing movements have been captured.

For the point-to-point movements, the participant was seated in front of a vertical canvas at a distance of 50 % of their arm length (see Figure 1). Four different targets appeared randomly on a vertical line on the canvas and the subjects were instructed to follow the targets fast, but precisely with their dominant hand. The vertical distance between the target positions varied between approximately 15 cm for small movements and 45 cm for large movements (distance between circle centers).

1.3 Experimental setup: Oscillation movements

For the oscillation movements, the participant was standing upright, holding the swing rod in their dominant hand and instructed to get the swing rod in resonance. Here, we additionally recorded the muscle surface electromyograms (EMG) of the m.bisceps and m.triceps muscle.

1.4 Experimental results: Point-to-point movements

The point-to-point movement of interest was repeated seven times. We extracted the different initial and target angles, as well as one intermittent posture which represents the point of maximal elbow angle (Table 2). These joint configurations were assumed to represent the equilibrium points for the neuronal controller in the simulation. See main manuscript for more details. The resulting trajectories in comparison to the experimental trajectories are shown in Figure 2.

initial angle		intermittant angle		target angle	
elbow	shoulder	elbow	shoulder	elbow	shoulder
66,86°	76,03°	83,30°	76,03°	72,02°	39,91°
62,73°	78,20°	78,54°	78,26°	69,08°	40,52°
63,08°	76,44°	78,99°	76,45°	69,56°	36,36°
61,06°	76,44°	76,61°	76,45°	67,92°	37,28°
60,47°	74,67°	77,53°	74,67°	70,39°	34,88°
61,53°	74,42°	80,79°	74,43°	68,77°	33,94°
60,18°	73,26°	76,74°	73,26°	67,79°	33,75°

Table 2: Equilibrium point angles extracted from the experimental data. All angles are given in degrees.

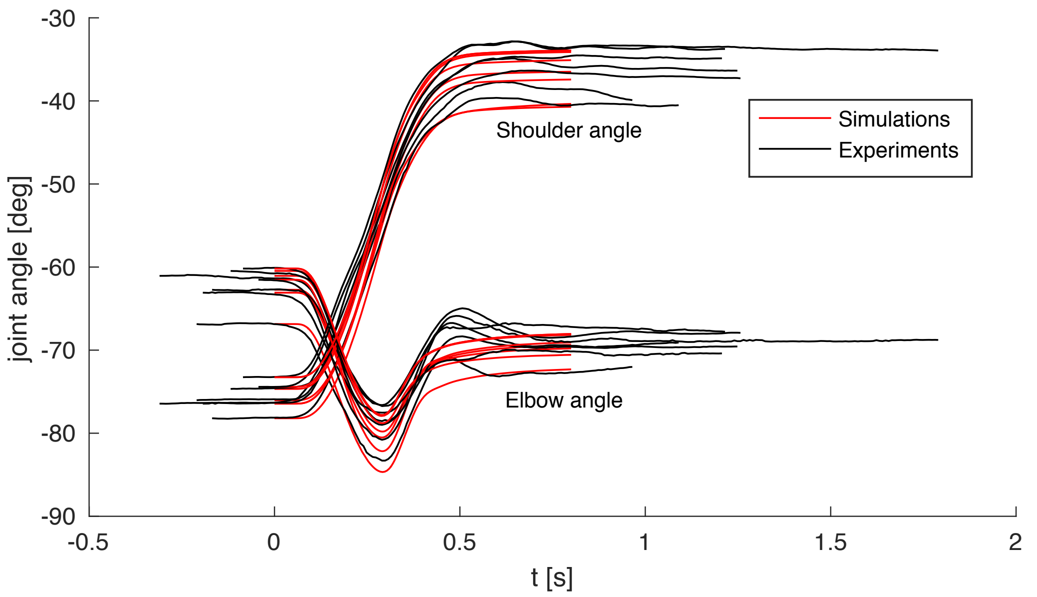

Figure 2: Variability of experimental data (black) and simulated data (red). These variations are the source of the error bar in the main manuscript.

1.5 Experimental results: Oscillation movements

The experimental data of the joint angles and EMG was analyzed with a fast Fourier analysis (Figure 3). This data indicates that the range of oscillation frequencies occurring in the movement is about ±0.2Hz. This was used as the variance of randomly selected frequencies to introduce a "natural" variation in the movements in the simulation.

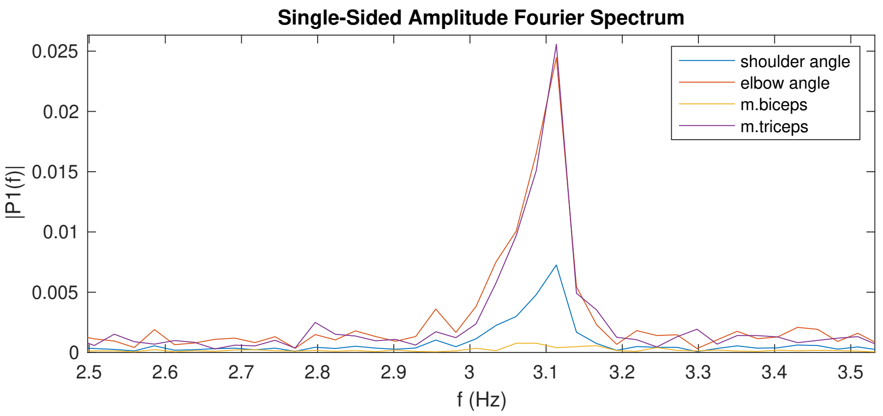

Figure 3: Fourier transformation of the experimental data on a swing rod exercise. Shown are the frequency spectra for two muscles (EMG of m. biceps and m.triceps) as well as the two joints (shoulder and elbow). This data indicates that the range of oscillation frequencies occurring in the movement is about ±0.2Hz. This was used as the variance of randomly selected frequencies to introduce a "natural" variation into the movements in the simulation.

2 Supplementary simulation results point-to-point movements

We further quantified the other three pointing movements, which have quite a different amplitude (and direction) than the point-to-point movement reported in the main manuscript. Despite these differences in movement, the main trend of the results is similar: morphological computation is highest for the highest level in the control hierarchy.

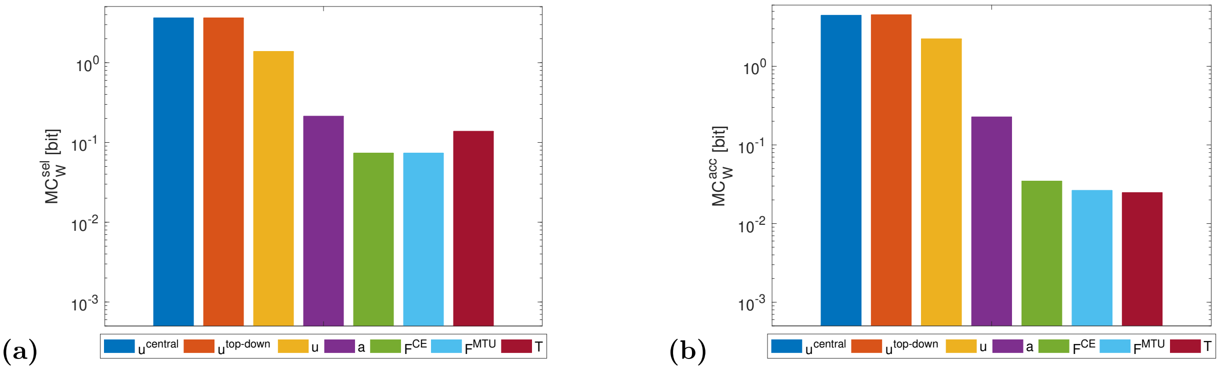

Figure 4: Morphological computation MC_W on different hierarchy levels for an exemplary point-to-point movement ($2 \rightarrow 3$). Morphological computation was evaluated using **(a)** selected (MC_W^{sel}) and **(b)** accumulated hierarchy levels (MC_W^{acc}). Note that a logarithmic scale is used for the y-axis. The limit of the y-axis is set to the maximum MC value that would result from having a constant signal as input.

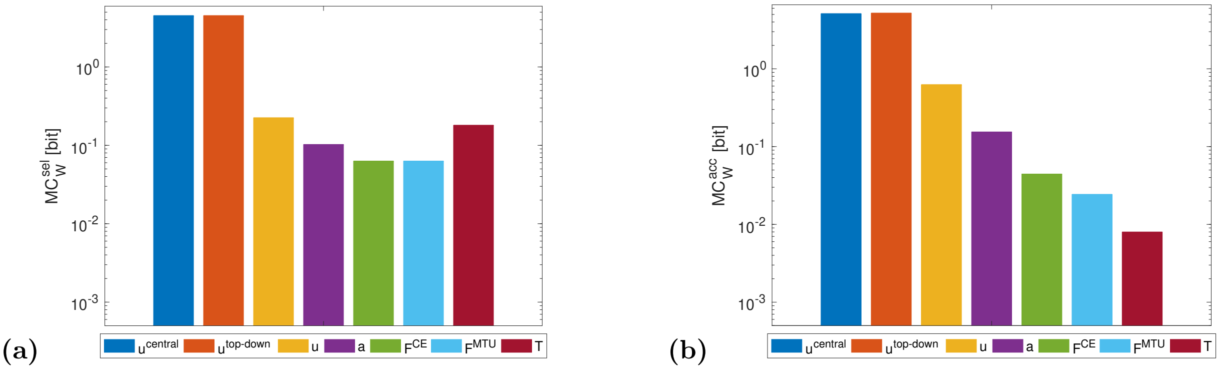

Figure 5: Morphological computation MC_W on different hierarchy levels for an exemplary point-to-point movement ($4 \rightarrow 1$). Morphological computation was evaluated using **(a)** selected (MC_W^{sel}) and **(b)** accumulated hierarchy levels (MC_W^{acc}). Note that a logarithmic scale is used for the y-axis. The limit of the y-axis is set to the maximum MC value that would result from having a constant signal as input.

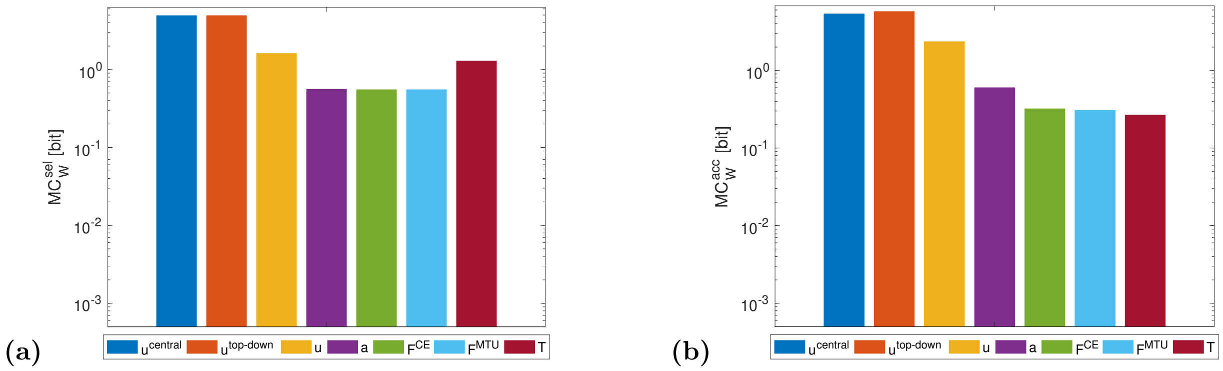

Figure 6: Morphological computation MC_W on different hierarchy levels for an exemplary point-to-point movement $(4 \rightarrow 3)$. Morphological computation was evaluated using **(a)** selected (MC_W^{sel}) and **(b)** accumulated hierarchy levels (MC_W^{acc}). Note that a logarithmic scale is used for the y-axis. The limit of the y-axis is set to the maximum MC value that would result from having a constant signal as input.

3 More details on quantifying morphological computation

This section discusses one concept of quantifying morphological computation. For a full discussion, the reader is referred to (Ghazi-Zahedi, 2019b).

3.1 Causal Model of the Sensorimotor Loop

We assume that there is a canonical way to separate a cognitive system into four parts, namely brain, sensors, actuators, and body. We are fully aware that the system–environment separation is a very difficult and yet unsolved question for biological systems (see e.g., (von Förster, 2003) for a discussion). In fact, one question of this paper is to investigate different levels of hierarchy with respect to quantifying morphological computation.

In our concept of the sensorimotor loop, which is derived from Pfeifer et al. (2007), the brain or controller sends signals to the actuators that influence the environment (see Figure 7). We prefer the notion of the system's *Umwelt* von Uexkuell (1957); Clark (1996); Ay and Löhr (2015), which is the part of the system's environment that can be affected by the system and itself affects the system. The state of the actuators and the *Umwelt* are not directly accessible to the cognitive system, but the loop is closed as information about both the *Umwelt* and the actuators are provided to the controller by the system's sensors. In addition to this general concept, which is widely used in the embodied artificial intelligence community (see e.g., (Pfeifer et al., 2007)), we introduce the notion of *world* to the sensorimotor loop, that is, the system's morphology and the system's *Umwelt*. This differentiation between body and world is analogous to the agent–environment distinction made in the context of reinforcement learning Sutton and Barto (1998), where the environment is defined as everything that cannot be changed arbitrarily by the agent.

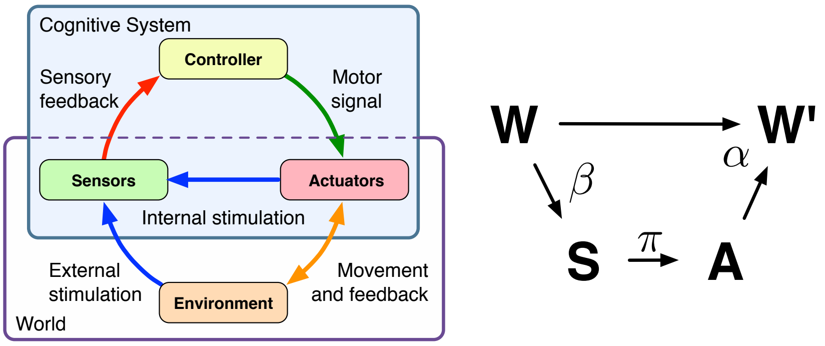

Figure 7: Sensorimotor Loop. Left-hand side: schematics of the sensorimotor loop (redrawn from Pfeifer et al. (2007)), Right-hand side: causal diagram of a reactive system.

Arm movements, and actually most behaviours that are interesting in the context of morphological computation, can be modelled sufficiently as reactive behaviours. Hence, for the remainder of this work, we will omit the controller and assume that the sensors are directly connected to the actuators. For a discussion of the causal diagram for non-reactive systems, the reader is referred to Ay and Zahedi (2014). Quantifications of morphological computation for non-reactive systems are discussed in Zahedi and Ay (2013).

The causal diagram of the sensorimotor loop is shown on the right-hand side of Figure 7. The random variables A, S, W, and W' refer to actuator signals, sensor signals, and the current and next world state. Directed edges reflect causal dependencies between the random variables. The random variables S and A are not to be mistaken with the sensors and actuators. The variable S is the output of the sensors, which is available to the controller or brain, and the action A is the input that the actuators take. Consider an artificial robotic system as an example. Then the sensor state S could be the pixel matrix delivered by a camera sensor

and the action A could be a numerical value that is taken by a motor controller and converted in currents to drive a motor.

We use capital letters (A, W, ...) to denote random variables, non-capital letters (a, w, ...) to denote a specific value that a random variable can take, and calligraphic letters ($\mathcal{A}$, $\mathcal{W}$, ...) to denote the alphabet for the random variables. This means that a_t is the specific value that the random variable A can take at time $t \in \mathbb{N}$, and it is from the set $a_t \in \mathcal{A}$. Greek letters refer to generative kernels, i.e., kernels that describe an actual underlying mechanism or a causal relation between two random variables. In the causal graphs throughout this paper, these kernels are represented by direct connections between corresponding nodes. This notation is used to distinguish generative kernels from others, such as the conditional probability of a given that w was previously seen, denoted by $p(a|w)$, which can be calculated or sampled but that does not reflect a direct causal relation between the two random variables A and W (see Figure 7).

We abbreviate the random variables for better comprehension in the remainder of this work, as all measures consider random variables of consecutive time indices. Therefore, we use the following notation. Random variables without any time index refer to time index t and hyphened variables to time index $t+1$. The two variables W and W' refer to W_t and W_{t+1}, respectively.

3.2 Quantifying Morphological Computation

We can now restate the two original concepts of quantifying morphological computation Zahedi and Ay (2013); Ghazi-Zahedi (2019b) (see Figure 8).

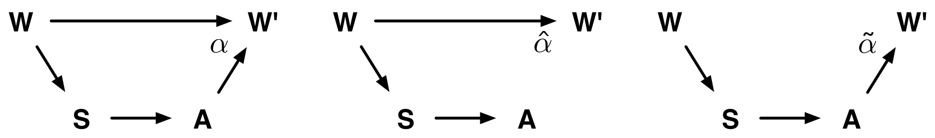

Figure 8: Visualisation of the two concepts $\mathrm{MC_A}$ and $\mathrm{MC_W}$. Left-hand side: causal diagram for a reactive system. Centre: causal diagram assuming no effect of the action A on the next world state W'. Right-hand side: causal diagram assuming no effect of the previous world state W on the next world state W'.

The basis for both original concepts $\mathrm{MC_A}$ and $\mathrm{MC_W}$ is the world dynamics kernel $\alpha(w'|w,a)$, which describes how the next world states W' depends on the current world state W and the current action A (see Figure 7, right-hand side, and Figure 8, left-hand side, respectively). For the first concept $\mathrm{MC_A}$, let us assume that there is no dependence of the next world state W' on the current action A. In this case, the world dynamics kernel $\alpha(w'|w,a)$ reduces to $\hat{\alpha}(w'|w)$ (which is given by $\hat{\alpha}(w'|w) = \sum_a {}^{p(w',w,a)}/_{p(w)}$, see also Figure 8, centre). As a result, we would state that we have maximal morphological computation, as the system's behaviour is not controlled by the brain at all. An example of such a system is the Passive Dynamic Walker (McGeer, 1990; Collins et al., 2005). We can measure how much the observed behaviour differs from this assumption with the Kullback-Leibler divergence (Cover and Thomas, 2006). This leads to the following formalisation:

$$\mathrm{MC_A} := \sum_{w',w,a} p(w',w,a) \log_2 \frac{\alpha(w'|w,a)}{\hat{\alpha}(w'|w)} \quad (1)$$

$$= I(W';A|W) \quad (2)$$

Unfortunately, Equation (1) is zero for maximal morphological computation, which is why we initially chose

to normalise and invert it, leading to the following definition:

$$\mathrm{MC_A} := 1 - \frac{1}{\log_2 |\mathcal{W}|} \sum_{w',w,a} p(w',w,a) \log_2 \frac{\alpha(w'|w,a)}{\hat{\alpha}(w'|w)} \tag{3}$$

The second concept, $\mathrm{MC_W}$ starts with the opposite assumption, namely, that the current world state W does not have any influence on the next world state W' (see Figure 8, right-hand side). In this case, the world dynamics kernel $\alpha(w'|w,a)$ reduces to $\tilde{\alpha}(w'|a)$ (which is given by $\tilde{\alpha}(w'|a) = \sum_w p(w',w,a)/p(a)$, see also Figure 8) and analogously to the following definition for $\mathrm{MC_W}$:

$$\mathrm{MC_W} := \sum_{w',w,a} p(w',w,a) \log_2 \frac{\alpha(w'|w,a)}{\tilde{\alpha}(w'|a)} \tag{4}$$

$$= I(W';W|A) \tag{5}$$

The relation of the measures to transfer entropy Schreiber (2000); Bossomaier et al. (2016) and the information bottleneck Tishby et al. (1999) are discussed in Zahedi and Ay (2013); Ghazi-Zahedi (2019b).

Next, we briefly describe, how the quantification can be calculated from data.

3.3 Estimating Quantifications of Morphological Computation

We will explain how to estimate information-theoretic quantities based on an estimation of entropy. A more detailed discussion about the topic can be found in (Paninski, 2003) and a more detailed discussion with respect to quantifying morphological intelligence can be found in (Ghazi-Zahedi, 2019b).

The general concepts will be discussed along the example of estimation entropy:

$$\hat{H}(X) = -\int_x \mu(x) \ln \mu(x) dx. \tag{6}$$

Estimating the entropy $H(X)$ requires knowledge about the underlying probability distribution $\mu(x)$. In real-world scenarios, $\mu(x)$ cannot be accessed directly, but only via observations x_i. The task is to estimate $p(x_i)$ such that the estimated entropy $H(X) = -\sum_x p(x) \ln p(x)$ approximates $\hat{H}(X)$. The most common technique is known as binning of frequency estimation. This method uses a discrimination of the state space X and estimates $p(x_i)$ by the number of samples that fall into each bin. Hence, the entropy of X is given by:

$$H(X) = -\sum_x \frac{c_i}{N} \ln \frac{c_i}{N}, \tag{7}$$

where c_i are the number of samples that fall into bin i and N is the total number of samples.

The second method is only discussed conceptually. For a full discussion, please read (Ghazi-Zahedi, 2019b; Kraskov et al., 2004; Frenzel and Pompe, 2007). The general idea is to estimate $p(x_i)$ without the intermediate step of binning the state space. Instead, the probability density is estimated by the distance to the k-nearest neighbour of each sample x_i. The distance to the k-nearest neighbour is small if the sample x_i is an area of the state space that has a high concentration of samples, and hence, the probability mass for x_i should be large. The distance to the k-nearest neighbour is large if the sample x_i is in a sparsly populated area of the state space. These assumptions are used to shape locally constant or Gaussian functions around each sample x_i.

Both methods can be used in *gomi* (Ghazi-Zahedi, 2019a) to estimate $\mathrm{MC_W}$ and $\mathrm{MC_A}$.

4 The arm model *Arm26*

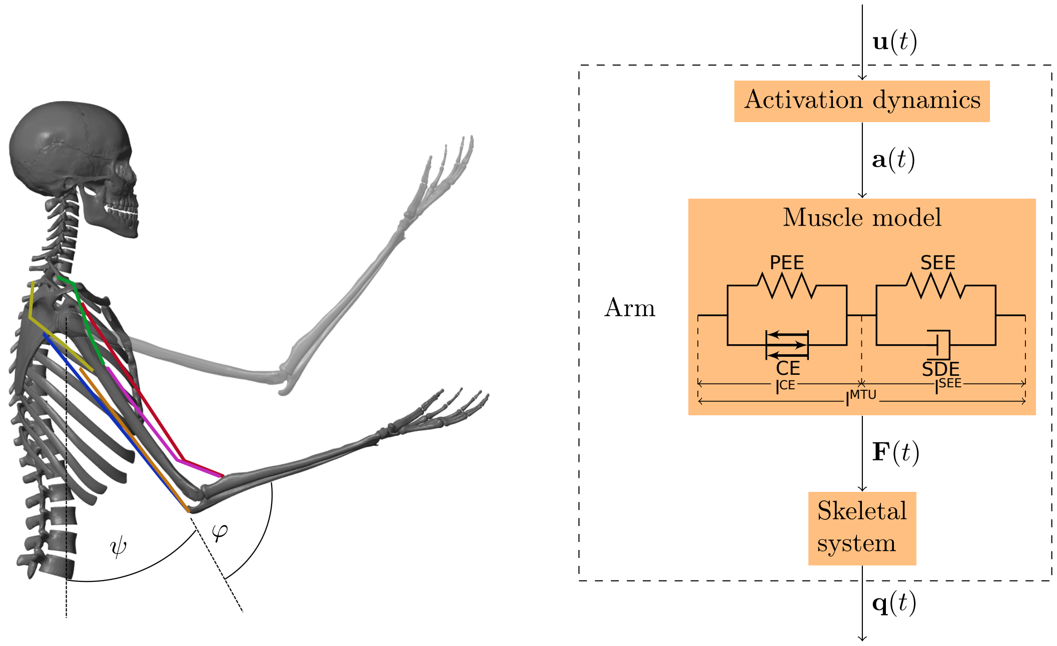

(a) (b)

Figure 9: (a) Visualization of the musculoskeletal model of the arm and the definition of the shoulder angle $\psi(t)$ and the elbow angle $\varphi(t)$ and (b) Structure of the arm model: the motor command $\mathbf{u}(t)$ is fed into the model of the activation dynamics of muscles which relates the neuronal stimulation to muscular activity $\mathbf{a}(t)$ that drives the muscle model. The muscles produce forces $\mathbf{F}(t)$ that act on the skeletal system resulting in a simulated movement $\mathbf{q}(t) = [\varphi(t), \psi(t)]$ of the arm.

The neuro-musculoskeletal model *Arm26* consists of a musculoskeletal model of the arm with two degrees of freedom actuated by six muscles and a controller. The model is implemented using Matlab® R2018a/Simulink® with the Simscape Multibody™environment. For a better overview, the implementation of the model is divided into three parts: the mechanical part (representing the bone structure and the muscle routing), the actuation of this mechanical part (muscle-tendon structures) and the controller (nervous system) which provides the input to the actuation part.

4.1 Musculoskeletal model of the arm: Mechanics and Actuation

The musculoskeletal model *Arm26* of the human arm uses the same geometry and muscle parameters as the simulation model described in Driess et al. (2018) which is based on Bayer et al. (2017). It consists of two rigid bodies (lower and upper arm) that are connected via two one-degree-of-freedom revolute joints that represent the shoulder and elbow joint. This multibody system is actuated by six muscle-tendon units (MTU), four monoarticular and two biarticular muscles (see Figure 9a). The muscles are modeled as lumped muscles, i.e. they represent a multitude of anatomical muscles:

1. Monarticular Elbow Flexor (MEF) (short: Elbow Flexor (EF)):
   m. brachioradialis, m. brachialis, m. pronator teres, m. extensor carpi radialis

2. Monarticular Elbow Extensor (MEE) (short: Elbow Extensor (EE)):
   m. triceps lateralis, m.triceps medialis, m. an-coneus, m. extensor carpi ulnaris

3. Biarticular Elbow Flexor Shoulder Anteversion (BEFSA) (short: Biarticular Flexor (BF)):
   m. biceps brachii caput longum and caput breve

4. Biarticular Elbow Extensor Shoulder Retroversion (BEESR) (short: Biarticular Extensor (BE)):
   m. triceps brachii caput longum

5. Monoarticular Shoulder Anteversion (MSA) (short: Shoulder Flexor (SF)):
   m. deltoideus (pars clavicularis, anterior, lateral), m. superior pectoralis major, m. coracobrachialis

6. Monoarticular Shoulder Retroversion (MSR) (short: Shoulder Extensor (SE)):
   m. deltoideus (pars spinalis, posterior), m. latissimus dorsi

The MTU structure is modeled using an extended Hill-type muscle model as described in Haeufle et al. (2014) with muscle activation dynamics as introduced by Hatze (1977). The muscle model is a macroscopic model consisting of four elements: the contractile element (CE), the parallel elastic element (PEE) and the serial elastic element (SEE) and serial damping element (SDE), as illustrated in Figure 9b. The inputs to the muscle model are the length of the MTU l^{MTU}, the contraction velocity of the MTU $\dot{l}^{\text{MTU}}$ and the muscular activity a. The output of the muscle model is a one-dimensional muscle force F^{MTU}. This force drives the movement of the skeletal system. For the routing of the muscle path around the joints, deflection ellipses are implemented as described by Hammer et al. (2019) (see Figure 10). The muscle path can move within these ellipses and is deflected as soon as it touches the boundary.

All in all, the governing model dependencies for all muscles $i = 1, ..., n$ are:

$$\dot{l}_i^{\text{CE}} = f_{\text{CE}}(l_i^{\text{CE}}, l_i^{\text{MTU}}, \dot{l}_i^{\text{MTU}}, a_i) \quad (8)$$

$$\dot{a}_i = f_a(a_i, u_i, l_i^{\text{CE}}) \quad (9)$$

$$F_i^{\text{MTU}} = f_F(l_i^{\text{MTU}}, \dot{l}_i^{\text{MTU}}, l_i^{\text{CE}}, a_i) \quad (10)$$

$$\ddot{\mathbf{q}} = f_q(\dot{\mathbf{q}}, \mathbf{q}, \mathbf{F}^{\text{MTU}}), \quad (11)$$

where $\mathbf{q}$ denotes a generalized state vector, in this case it can be defined as $\mathbf{q} = [\varphi, \psi]$ and $\mathbf{F}^{\text{MTU}} = \{F_i^{\text{MTU}}\}_{i=1}^{n}$.

The mechanical parameters of the arm segments are taken from Kistemaker et al. (2006) and can be found in Table 3. The positions and sizes of the deflection ellipses were chosen in order to match moment arms in literature (see Figure 11) and can be found in Listing 1. For more details on this see Suissa (2017). The (non-)muscle-specific parameters can be found in Table 4 and Table 5.

	Length [m]	d [m]	Mass [kg]	I [kgm^2]
Upper arm	0.335	0.146	2.10	0.024
Lower arm	0.263	0.179	1.65	0.025

Table 3: Mechanical parameters of the skeletal structure (Kistemaker et al. (2006)) with d: distance from proximal joint to center of mass and I: moment of inertia with respect to the center of mass.

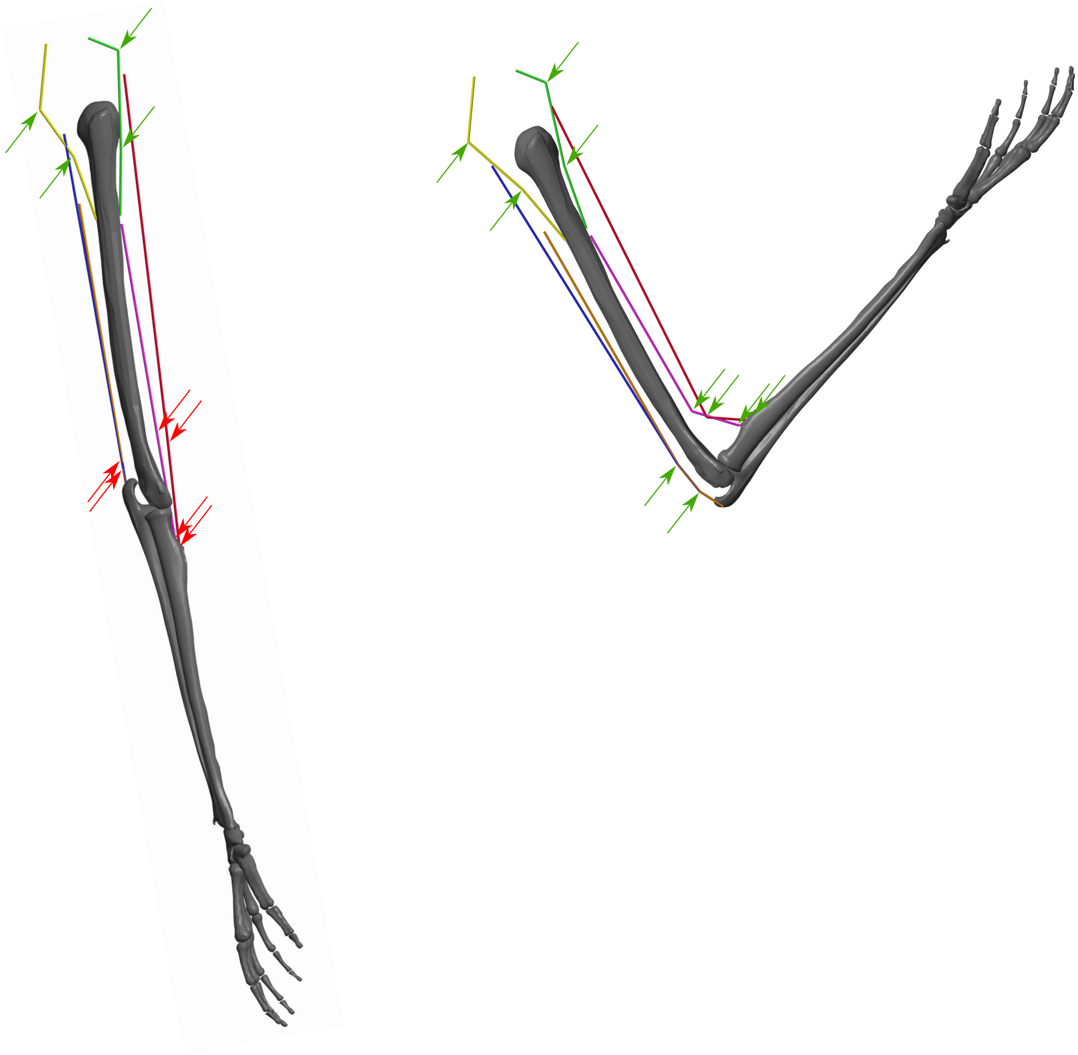

Figure 10: Illustration of the positions of the deflection ellipses that are used for the muscle routing in two different arm positions. Green arrows indicate active ellipses that deflect the muscle path, while red arrows indicate inactive ellipses that do not change the muscle path.

	$F^{\max}$ [N]	$l^{\text{CE,opt}}$ [m]	$l^{\text{SEE,0}}$ [m]
Monarticular Elbow Flexor (MEF)	1420	0.092	0.182
Monarticular Elbow Extensor (MEE)	1550	0.093	0.187
Monoarticular Shoulder Anteversion (MSA)	838	0.134	0.039
Monoarticular Shoulder Retroversion (MSR)	1207	0.140	0.066
Biarticular Elbow Flexor Shoulder Anteversion (BEFSA)	414	0.151	0.245
Biarticular Elbow Extensor Shoulder Retroversion (BEESR)	603	0.152	0.260

Table 4: Muscle-specific actuation parameters (Kistemaker et al. (2006) and Kistemaker et al. (2013)), with $F^{\max}$: maximum isometric force, $l^{\text{CE,opt}}$: optimal length of the contractile element, $l^{\text{SEE,0}}$ rest length of the serial elastic element. The lengths of $l^{\text{CE,opt}}$ and $l^{\text{SEE,0}}$ were adapted to match the muscle path routed through the ellipses in order to allow for a big range of motion. For this parameter adaptation method see Suissa (2017).

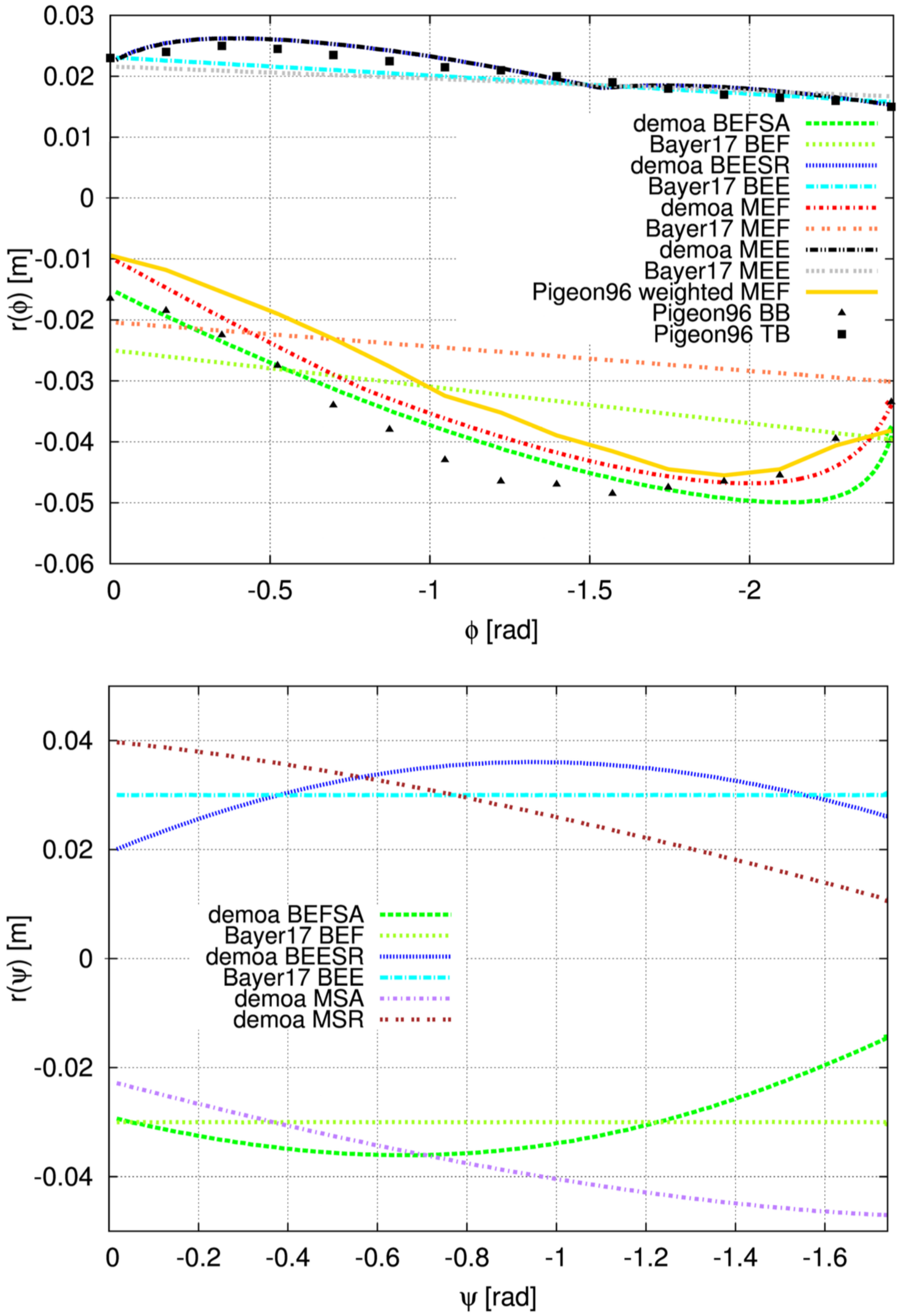

Figure 11: Comparison of the moment arms of the muscles in the model with simulation and experimental data from literature for the elbow muscles (upper plot) and the shoulder muscles (lower plot). The lines marked with "demoa" refer to the model by Suissa (2017) on which our model is based (for the naming of the muscles see Table 4). We use the same geometry and position and size of the ellipses, so our moment arms are the same as in the "demoa" model. The moment arms are compared to a calculatory model by Bayer et al. (2017) (here M/B stands for mono- and biarticular, E stands for elbow and F/E stands for flexion and extension, respectively) and to experimental data. The black marks show experimental data of the biceps brachii (BB) and the triceps brachii (TB) taken from Pigeon et al. (1996). The yellow line shoes a weighted combination of the monoarticular flexor muscles that are represented by the MEF in the model. They are weighted according to their proportion of the joint torques, see Sobotta (2010); Aumüller et al. (2017). The figure was taken from Suissa (2017) with kind permission of the author.

	Parameter	Unit	Value	Source	Description
CE	ΔW^{des}	[ ]	0.45	similar to Bayer et al. (2017); Kistemaker et al. (2006)	width of normalized bell curve in descending branch, adapted to match observed force-length curves
	ΔW^{asc}	[ ]	0.45	similar to Bayer et al. (2017); Kistemaker et al. (2006)	width of normalized bell curve in ascending branch, adapted to match observed force-length curve
	$\nu^{\mathrm{CE,des}}$	[ ]	1.5	Mörl et al. (2012)	exponent for descending branch
	$\nu^{\mathrm{CE,asc}}$	[ ]	3.0	Mörl et al. (2012)	exponent for ascending branch
	$A^{\mathrm{rel,0}}$	[ ]	0.2	Günther (1997)	parameter for contraction dynamics: maximum value of A^{rel}
	$B^{\mathrm{rel,0}}$	[1/s]	2.0	Günther (1997)	parameter for contraction dynamics: maximum value of B^{rel}
	$\mathcal{S}^{\mathrm{ecc}}$	[ ]	2.0	van Soest et al. (1993)	relation between $F(v)$ slopes at $v^{\mathrm{CE}} = 0$
	$\mathcal{F}^{\mathrm{ecc}}$	[ ]	1.5	van Soest et al. (1993)	factor by which the force can exceed F^{isom} for large eccentric velocities
PEE	$\mathcal{L}^{\mathrm{PEE,0}}$	[ ]	0.95	Günther (1997)	rest length of PEE normalized to optimal length of CE
	ν^{PEE}	[ ]	2.5	Mörl et al. (2012)	exponent of F^{PEE}
	$\mathcal{F}^{\mathrm{PEE}}$	[ ]	2.0	Mörl et al. (2012)	force of PEE if l^{CE} is stretched to ΔW^{des}
SDE	D^{SDE}	[ ]	0.3	Mörl et al. (2012)	dimensionless factor to scale $d^{\mathrm{SDE,max}}$
	R^{SDE}	[ ]	0.01	Mörl et al. (2012)	minimum value of d^{SDE} (at $F^{MTU} = 0$), normalized to $d^{\mathrm{SDE,max}}$
SEE	$\Delta U^{\mathrm{SEE,nll}}$	[ ]	0.0425	Mörl et al. (2012)	relative stretch at non-linear linear transition
	$\Delta U^{\mathrm{SEE,l}}$	[ ]	0.017	Mörl et al. (2012)	relative additional stretch in the linear part providing a force increase of $\Delta F^{\mathrm{SEE,0}}$
	$\Delta F^{\mathrm{SEE,0}}$	[N]	$0.4\,F^{\mathrm{max}}$		both force at the transition and force increase in the linear part
Hatze	m	[1/s]	11.3	Kistemaker et al. (2006)	time constant for the activation dynamics
	c	[mol/l]	1.37e-4	Kistemaker et al. (2006)	constant for the activation dynamics
	η	[l/mol]	5.27e4	Kistemaker et al. (2006)	constant for the activation dynamics
	k	[ ]	2.9	Kistemaker et al. (2006)	constant for the activation dynamics
	q_0	[ ]	0.005	Günther (1997)	resting active state for all activated muscle fibers
	ν	[ ]	3	Kistemaker et al. (2006)	constant for the activation dynamics

Table 5: Muscle non-specific actuation parameters for the muscles and the activation dynamics.

```
% Gravity
PM.Gravity = [0 0 -9.80665];

%% %%%%%%%%%%%%%%%%%%%%
% Segment parameters %
%%%%%%%%%%%%%%%%%%%%%%
PM.SegShoulder.p_Bone_CoM           = [0 0 0]; %[m] position of Bone CoM relative to proximal joint
PM.SegShoulder.p_joint_distal       = [0 -.1816 0]; %[m] position of distal joint relative to CoM

PM.SegShoulder.m_Bone               = 16.895560; %[kg] m
PM.SegShoulder.MomInert_Bone        = [0.096243 0.0811621 0.159251]; %[kg*m^2] Diagonal elements of the inertia tensor
PM.SegShoulder.ProdInert_Bone       = [0 0 0]; %[kg*m^2] Non-diagonal elements of the intertia tensor in the order [I_yz I_zx I_xy]
PM.SegUparm.p_Bone_CoM              = [0 0 0.146]*(-1); %[m] position of Bone CoM relative to proximal joint % *(-1) in comparison with demoa, source: Kistemaker2007
PM.SegUparm.p_joint_distal          = [0 0 -0.189]; %[m] position of distal joint relative to CoM

PM.SegUparm.m_Bone                  = 2.10; %[kg] mass, source: Kistemaker2007
PM.SegUparm.MomInert_Bone           = [0.0154388 0.024 0.00278951]; %[kg*m^2] Diagonal elements of the inertia tensor
PM.SegUparm.ProdInert_Bone          = [0 0 0]; %[kg*m^2] Non-diagonal elements of the intertia tensor in the order [I_yz I_zx I_xy]
PM.SegForearm.p_Bone_CoM            = [0 0 0.179]*(-1); %[m] position of Bone CoM relative to proximal joint, source: Kistemaker2007
PM.SegForearm.p_joint_distal        = [0 0 -0.084]; %[m] position of distal joint relative to CoM %Note: difference to demoa model, where: [0.015 0 -0.084]

PM.SegForearm.m_Bone                = 1.65; %[kg] mass, source: Kistemaker2007
PM.SegForearm.MomInert_Bone         = [0.009824518 0.025 0.001500813]; %[kg*m^2] Diagonal elements of the inertia tensor
PM.SegForearm.ProdInert_Bone        = [0 0 0]; %[kg*m^2] Non-diagonal elements of the intertia tensor in the order [I_yz I_zx I_xy]
PM.SegHand.p_Bone_CoM               = [0 0 0.07]*(-1); %[m] position of Bone CoM relative to proximal joint %Note: difference to demoa model, where: [0.015 0.0000 0.07]*(-1)
PM.SegHand.p_fingertip              = [0 0 -0.084]; %[m] position of distal joint relative to CoM

PM.SegHand.m_Bone                   = 0; %[kg] m
PM.SegHand.MomInert_Bone            = [0 0 0]; %[kg*m^2] Diagonal elements of the inertia tensor
PM.SegHand.ProdInert_Bone           = [0 0 0]; %[kg*m^2] Non-diagonal elements of the intertia tensor in the order [I_yz I_zx I_xy]

%%%%%%%%%%%%%%%%%%%%%%
% Deflection parameters %
%%%%%%%%%%%%%%%%%%%%%%

PM.Deflection.biart_flexor.rO                       = [0.03 -0.1816 0.02]; % Origin of the muscle relative to the center of mass of the parent body %Parent: Shoulder
PM.Deflection.biart_flexor.rI                       = [0.012, 0.0000, 0.12]; % Insertion of the muscle %Parent: Forearm
PM.Deflection.biart_flexor.Ellipse1.r               = [0.018, 0.0000, -0.1425]; % Coordinates of the reference point of deflection ellipse 1 relative to the parent body %Parent:Uparm
PM.Deflection.biart_flexor.Ellipse1.G               = [0 1 0]*0.0001; % Length of the half-axis of ellipse 1 in y direction
PM.Deflection.biart_flexor.Ellipse1.H               = [0 0 1]*0.0001; % % Length of the half-axis of ellipse 1 in z direction
PM.Deflection.biart_flexor.Ellipse1.angle           = [0,90,0]; % Angle [deg] of rotation of the ellipse triade around y-axis to orient the ellipse correctly
PM.Deflection.biart_flexor.Ellipse2.r               = [0.012, 0.0000, 0.125]; %Parent: Forearm
PM.Deflection.biart_flexor.Ellipse2.G               = [0 1 0]*0.0001;
PM.Deflection.biart_flexor.Ellipse2.H               = [0 0 1]*0.0001;
PM.Deflection.biart_flexor.Ellipse2.angle           = [0,0,0];

PM.Deflection.biart_extensor.rO                     = [-0.02 -0.1816 -0.03]; %Parent: Shoulder
PM.Deflection.biart_extensor.rI                     = [-0.0225, 0.0000, 0.1925]; %Parent: Forearm
PM.Deflection.biart_extensor.Ellipse1.r             = [-0.0225, 0.0000, -0.165]; %Parent:Uparm
PM.Deflection.biart_extensor.Ellipse1.G             = [0 1 0]*0.0001;
PM.Deflection.biart_extensor.Ellipse1.H             = [0 0 1]*0.00001;
PM.Deflection.biart_extensor.Ellipse1.angle         = [0,-90,0];
PM.Deflection.biart_extensor.Ellipse2.r             = [-0.0, 0.0000, 0.1975]; %Parent: Forearm
PM.Deflection.biart_extensor.Ellipse2.G             = [0 1 0]*0.0001;
PM.Deflection.biart_extensor.Ellipse2.H             = [0 0 1]*0.03;
PM.Deflection.biart_extensor.Ellipse2.angle         = [0,-60,0];

PM.Deflection.Shoulder_Anteversion.rO               = [0.00, -0.1816, 0.05]; %Parent: Shoulder
PM.Deflection.Shoulder_Anteversion.rI               = [0.01, 0.0000, 0.045];  %Parent:Uparm
PM.Deflection.Shoulder_Anteversion.Ellipse1.r       = [0.025, -0.1816, 0.04]; %Parent: Shoulder
PM.Deflection.Shoulder_Anteversion.Ellipse1.G       = [0 1 0]*0.0001;
PM.Deflection.Shoulder_Anteversion.Ellipse1.H       = [0 0 1]*0.0001;
PM.Deflection.Shoulder_Anteversion.Ellipse1.angle   = [0,0,0];
PM.Deflection.Shoulder_Anteversion.Ellipse2.r       = [0.02, 0.0000, 0.1];  %Parent:Uparm
PM.Deflection.Shoulder_Anteversion.Ellipse2.G       = [0 1 0]*0.0001;
PM.Deflection.Shoulder_Anteversion.Ellipse2.H       = [0 0 1]*0.0001;
PM.Deflection.Shoulder_Anteversion.Ellipse2.angle   = [0,90,0];

PM.Deflection.Shoulder_Retroversion.rO              = [-0.035, -0.1816, 0.045]; %Parent: Shoulder
PM.Deflection.Shoulder_Retroversion.rI              = [-0.01, 0.0000, 0.045];  %Parent:Uparm
PM.Deflection.Shoulder_Retroversion.Ellipse1.r      = [-0.04, -0.1816, -0.01]; %Parent: Shoulder
PM.Deflection.Shoulder_Retroversion.Ellipse1.G      = [0 1 0]*0.0001;
PM.Deflection.Shoulder_Retroversion.Ellipse1.H      = [0 0 1]*0.0001;
PM.Deflection.Shoulder_Retroversion.Ellipse1.angle  = [0,0,0];
PM.Deflection.Shoulder_Retroversion.Ellipse2.r      = [-0.02, 0.0000, 0.1];  %Parent:Uparm
PM.Deflection.Shoulder_Retroversion.Ellipse2.G      = [0 1 0]*0.0001;
PM.Deflection.Shoulder_Retroversion.Ellipse2.H      = [0 0 1]*0.0001;
PM.Deflection.Shoulder_Retroversion.Ellipse2.angle  = [0,90,0];

PM.Deflection.Elbow_flexor.rO                       = [0.01, 0.0000, 0.038]; %Parent:Uparm
PM.Deflection.Elbow_flexor.rI                       = [0.01, 0.0000, 0.12]; %Parent: Forearm
PM.Deflection.Elbow_flexor.Ellipse1.r               = [0.0, 0.0000, -0.132]; %Parent:Uparm
PM.Deflection.Elbow_flexor.Ellipse1.G               = [0 1 0]*0.0001;
PM.Deflection.Elbow_flexor.Ellipse1.H               = [0 0 1]*0.005;
PM.Deflection.Elbow_flexor.Ellipse1.angle           = [0,90,0];
PM.Deflection.Elbow_flexor.Ellipse2.r               = [0.01, 0.0000, 0.135]; %Parent: Forearm
PM.Deflection.Elbow_flexor.Ellipse2.G               = [0 1 0]*0.0001;
PM.Deflection.Elbow_flexor.Ellipse2.H               = [0 0 1]*0.003;
PM.Deflection.Elbow_flexor.Ellipse2.angle           = [0,90,0];
```

```
PM.Deflection.Elbow_extensor.rO                    = [-0.022, 0.0000, 0.0605]; %Parent:Uparm
PM.Deflection.Elbow_extensor.rI                    = [-0.0225, 0.0000, 0.1925]; %Parent: Forearm
PM.Deflection.Elbow_extensor.Ellipse1.r            = [-0.0225, 0.0000, -0.165]; %Parent:Uparm
PM.Deflection.Elbow_extensor.Ellipse1.G            = [0 1 0]*0.0001;
PM.Deflection.Elbow_extensor.Ellipse1.H            = [0 0 1]*0.00005;
PM.Deflection.Elbow_extensor.Ellipse1.angle        = [0,-90,0];
PM.Deflection.Elbow_extensor.Ellipse2.r            = [-0.0, 0.0000, 0.1975]; %Parent: Forearm
PM.Deflection.Elbow_extensor.Ellipse2.G            = [0 1 0]*0.0001;
PM.Deflection.Elbow_extensor.Ellipse2.H            = [0 0 1]*0.03;
PM.Deflection.Elbow_extensor.Ellipse2.angle        = [0,-60,0];
```

Listing 1: Mechanics parameters defining the geometry and the mechanical properties.

4.2 Equilibrium Point Controller

The bio-inspired hybrid equilibrium point controller exploits muscle characteristics by combining a feed-forward command ($\mathbf{u}^{\text{open}}(t)$) with spinal feedback on muscle fiber lengths ($\mathbf{u}^{\text{closed}}(t)$). This feedback represents a simplified version of the mono-synaptic muscle spindle reflex, assuming that the muscle spindles provide accurate time-delayed information about the muscle fiber lengths $\mathbf{l}^{\text{CE}}(t)$ (Kistemaker et al., 2006). The total motor command u_i for each muscle i is a sum of those components and is calculated as

$$\begin{aligned} u_i(t) &= \left\{u_i^{\text{open}}(t) + u_i^{\text{closed}}(t) + u_i^{\text{CPG}}(t)\right\}_0^1 \\ &= \left\{u_i^{\text{open}}(t) + \frac{k_p}{l^{\text{CE,opt}}}\left(\lambda_i(t) - l_i^{\text{CE}}(t-\delta)\right) + u_i^{\text{CPG}}(t)\right\}_0^1 , \end{aligned} \tag{12}$$

where k_p is a feedback gain and the time delay δ is set to 10 ms representing a short-latency reflex delay which is in a physiologically plausible range (More et al., 2010; Houk and Rymer, 1981). $l^{\text{CE,opt}}$ stands for the optimal length of the contractile element. The operation $\{x\}_0^1$ sets values $x < 0$ to 0 and $x > 1$ to 1. The signal u_i^{CPG} represents a central pattern generator (CPG).

The low-level controller gets two top-down input signals: The open-loop muscle stimulation $u_i^{\text{open}}(t)$ and the desired muscle fiber lengths $\lambda_i(t)$. Here, they represent an intermittent control approach, because they are piecewise constant functions over time. Herein, each constant value represents an *equilibrium posture* (EP), i.e. the system is in a stable equilibrium in these positions:

$$\dot{\mathbf{q}} = 0 \quad \text{and} \quad \ddot{\mathbf{q}} = 0 , \tag{13}$$

leading to the condition that the net joint moment vanishes in these postures (given enough time for the system to settle). This allows for the calculation of the muscle stimulations $u_i^{\text{open}}(t)$ and the corresponding desired muscle fiber lengths $\lambda_i(t)$: For each EP, the muscle stimulations $u_i^{\text{open}}(t)$ can be determined by minimizing the difference between the muscle stimulation u_i^{open} and the desired level of co-contraction $u^{\text{des.}}$:

$$\sum_{i=1}^{4}(u_i^{\text{open}} - u^{\text{des.}}) \rightarrow \min , \tag{14}$$

subject to the constraint that the sum of all torques acting on the joint is zero, i.e. the system is in a stable equilibrium position. The corresponding desired muscle fiber lengths λ_i are set by measuring the length of the muscle fiber lengths l_i^{CE} in the equilibrium positions.

4.2.1 Optimization of the control parameters for a point-to-point movement

For the goal-directed point-to-point movements, three EPs were used per movement. To follow experimental trajectories, we optimized some of the control parameters: the shoulder and elbow angle for the second EP, the desired level of co-contraction for the second and the third EP, the starting times for the second and the third EP and the feedback gain k_p. Using the pattern search algorithm in Matlab®, the quadratic difference between the simulated and the experimental trajectory was minimized. The resulting parameters can be found in Table 6.

Using the resulting parameters, the muscle stimulations u_i^{open} were then optimized in order to fulfill the conditions for equilibrium points (see Equation 14) using the Matlab® optimizer *fmincon* which is suitable for finding the minimum of a constraint function.

		1 → 4	2 → 3	4 → 1	4 → 3
Shoulder Angle	EP 1	76.4°	60.4°	36.4°	40.8°
	EP 2	42.3°	37.5°	54.3°	47.1°
	EP 3	37.3°	46.7°	75.9°	48.4°
Elbow Angle	EP 1	61.1°	70.5°	70.0°	69.2°
	EP 2	74.3°	77.5°	76.0°	75.9°
	EP 3	67.9°	74.3°	61.9°	73.4°
Level of co-contraction	EP 1	0.10	0.10	0.10	0.10
	EP 2	0.077	0.094	0.38	0.33
	EP 3	0.28	0.20	0.5	0.44
Switching times	EP 1	0.00s	0.00s	0.00s	0.00s
	EP 2	0.35s	0.43s	0.37s	0.19s
	EP 3	0.57s	0.63s	0.57	0.41s
k_p		0.27	0.09	0.92	0.39

Table 6: Resulting control parameters for the computer simulation of point-to-point movements. Some simulation parameters resulted from the optimization that was performed to match the corresponding experimental trajectories: the shoulder and elbow angle for the second EP, the desired level of co-contraction for the second and the third EP, the starting times for the second and the third EP and the feedback gain k_p. The other parameters were set according to the experimental data.

4.3 Dynamic oscillation movements with vibrating rod

The dynamic oscillation movements were inspired by the training and rehabilitation exercises performed with a vibrating rod. When we experienced such a training, we noticed how, after some training, we were able to excite the resonance frequency of the rod. This seems an interesting task in the sense of morphological computation, as the dynamic interaction with the "umwelt" with its specific resonance characteristics, is surprisingly not that difficult to learn.

A concept for modeling rhythmic excitement of the processes is the concept of the central pattern generator (CPG). Central pattern generators are neural networks with the ability to produce rhythmic patterns without receiving rhythmic input signals from higher control centers or sensory feedback. The control concept of CPG implemented here is a simple sinusoidal pattern with a defined phase-shift between the stimultations to the extensor and flexor muscles. This was inspired by the work of Sproewitz et al. (2008).

The vibrating rod was modeled based on a therapy rod called “Propriomed 1" that is produced by the company *Haider Bioswing.* To implement the vibrating rod in Simulink the mechanical parameters of the spring-mass damper system are determinded experimentally including the mass m, the spring constant k and the damping constant d. For this purpose the vibrating rod was fixed and equipped with reflecting markers at both ends of the rod. The vibrating rod is then deflected evenly on both sides and the resulting vibration is recorded using a motion capture system(VICON motus). The resulting oscillation is analyzed and the mechanical properties are calculated with the formula of the quasiharmonic oscillation. Resulting in a damping constant of $d = 0,405\,\mathrm{kg/s}$ and a spring stiffness of $k = 300,523\,\mathrm{kg/s^2}$.

In order to verify the values for the spring and damper constant calculated from the experimental excitation the calculated parameters are fed into the Simulink model and a bilateral deflection is simulated.

The Simulink model of the vibrating rod is connected to the wrist and consists of a prismatic joint as well as

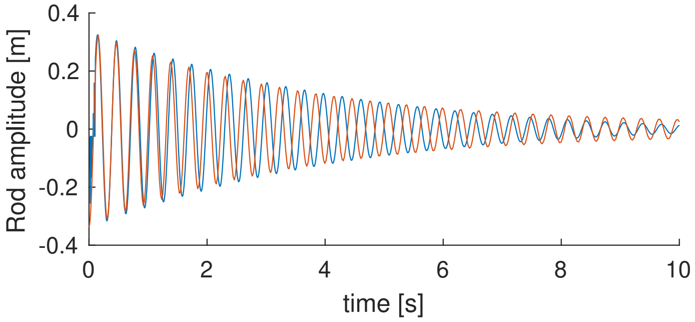

Figure 12: The oscillation generated in the simulation (in orange) corresponds to a large extent to the experimentally generated oscillation (in blue) at the beginning. Only after a few oscillations a deviation in amplitude and frequency can be observed.

the mass of the vibrating rod. The prismatic joint is actuated by a visco-elastic force element, which contains the spring and damper constants. The is guided by a virtual rail to limit the movement to horizontal forward movement, compensating for the influence of gravity.

The control of rhythmic movements is performed based on the general controller described in the main text

$$u_i(t) = \left\{ u_i^{\text{open}}(t) + \frac{k_p}{l^{\text{CE,opt}}} \left(\lambda_i(t) - l_i^{\text{CE}}(t-\delta)\right) + u_i^{\text{CPG}}(t) \right\}_0^1 , \tag{15}$$

The first two terms are parameterized such that, in the absence of a CPG signal, the hand holds the rod in front of the body at rest.

To excite the rod, as done in training and rehabilitation exercises, a sinusoidal signal $\mathbf{u}^{\text{CPG}}$ mimicking the output of a central pattern generator (CPG) is added to the motor command $\mathbf{u}$:

$$\mathbf{u}^{\text{CPG}}(t) = \hat{u} \cdot \sin(\omega \cdot t + \phi_0) , \tag{16}$$

with $\hat{u} = 0.1$: amplitude, ω: angular frequency, ϕ_0: phase. The muscles are synchronized by setting $\phi_0 = 0$ for flexing muscles and $\phi_0 = \pi$ for extending muscles.

The oscillation is exited for $0 \leq t \leq 4$s. After this, $\mathbf{u}^{\text{CPG}} = 0$ and the oscillation is then only a result of the dynamics of the system and not of the controller anymore.

List of Abbreviations

MTU muscle-tendon unit

CE contractile element

PEE parallel elastic element

SEE serial elastic element

SDE serial damping element

EF Elbow Flexor

MEF Monarticular Elbow Flexor

EE Elbow Extensor

MEE Monarticular Elbow Extensor

BF Biarticular Flexor

BEFSA Biarticular Elbow Flexor Shoulder Anteversion

BE Biarticular Extensor

BEESR Biarticular Elbow Extensor Shoulder Retroversion

SF Shoulder Flexor

MSA Monoarticular Shoulder Anteversion

SE Shoulder Extensor

MSR Monoarticular Shoulder Retroversion

References

Aumüller, G., Aust, G., Engele, J., Kirsch, J., Maio, G., and Mayerhofer, A. *Duale Reihe Anatomie.* 2017.

Ay, N. and Löhr, W. The umwelt of an embodied agent—a measure-theoretic definition. *Theory in Biosciences*, 134(3):105–116, 2015.

Ay, N. and Zahedi, K. On the causal structure of the sensorimotor loop. In Prokopenko, M., editor, *Guided Self-Organization: Inception*, volume 9 of *Emergence, Complexity and Computation.* Springer, 2014.

Bayer, A., Schmitt, S., Günther, M., and Haeufle, D. F. The influence of biophysical muscle properties on simulating fast human arm movements. *Computer Methods in Biomechanics and Biomedical Engineering*, 20(8):803–821, 2017.

Bossomaier, T., Barnett, L., Harré, M., and Lizier, J. T. *An Introduction to Transfer Entropy.* Springer, 2016.

Clark, A. *Being There: Putting Brain, Body, and World Together Again.* MIT Press, Cambridge, MA, USA, 1996.

Collins, S., Ruina, A., Tedrake, R., and Wisse, M. Efficient bipedal robots based on passive-dynamic walkers. *Science*, 307(5712):1082–1085, 2005.

Cover, T. M. and Thomas, J. A. *Elements of Information Theory*, volume 2nd. Wiley, Hoboken, New Jersey, USA, 2006.

Driess, D., Zimmermann, H., Wolfen, S., Suissa, D., Haeufle, D., Hennes, D., Toussaint, M., and Schmitt, S. Learning to Control Redundant Musculoskeletal Systems with Neural Networks and SQP: Exploiting Muscle Properties. *ICRA 2018 (accepted)*, 2018.

Frenzel, S. and Pompe, B. Partial mutual information for coupling analysis of multivariate time series. *Phys. Rev. Lett.*, 99:204101, 2007.

Ghazi-Zahedi, K. Gomi github repository. http://github.com/kzahedi/gomi, 2019a.

Ghazi-Zahedi, K. *Morphological Intelligence: Measuring the Body's Contribution to Intelligence.* Springer London, 2019b.

Günther, M. *Computersimulationen zur Synthetisierung des muskulär erzeugten menschlichen Gehens unter Verwendung eines biomechanischen Mehrkörpermodells.* PhD thesis, Eberhard-Karls-Universität zu Tübingen, 1997.

Haeufle, D. F. B., Günther, M., Bayer, A., and Schmitt, S. Hill-type muscle model with serial damping and eccentric force-velocity relation. *Journal of Biomechanics*, 47(6):1531–1536, 2014.

Hammer, M., Günther, M., Haeufle, D., and Schmitt, S. Tailoring anatomical muscle paths: a sheath-like solution for muscle routing in musculoskeletal computer models. *Mathematical Biosciences*, accepted (February):68–81, 2019. doi: 10.1016/j.mbs.2019.02.004.

Hatze, H. A Myocybernetic Control Model of Skeletal Muscle. *Biol. Cybernetics*, 25:103–119, 1977.

Houk, J. and Rymer, W. Neural control of muscle length and tension. In *Supplement 2. Handbook of Physiology, The Nervous System, Motor Control.* 1981.

Kistemaker, D. a., Van Soest, A. J., and Bobbert, M. F. Is equilibrium point control feasible for fast goal-directed single-joint movements? *Journal of Neurophysiology*, 95(5):2898–912, 2006.

Kistemaker, D. A., Van Soest, A. J. K., Wong, J. D., Kurtzer, I., and Gribble, P. L. Control of position and movement is simplified by combined muscle spindle and Golgi tendon organ feedback. *Journal of Neurophysiology*, 109(4):1126–1139, 2013.

Kraskov, A., Stögbauer, H., and Grassberger, P. Estimating mutual information. *Phys. Rev. E*, 69:066138, 2004.

McGeer, T. Passive walking with knees. In *In Robotics and Automation*, pages 1640–1645, 1990.

More, H. L., Hutchinson, J. R., Collins, D. F., Weber, D. J., Aung, S. K. H., and Donelan, J. M. Scaling of sensorimotor control in terrestrial mammals. *Proceedings of the Royal Society B: Biological Sciences*, 277 (1700):3563–3568, 2010.

Mörl, F., Siebert, T., Schmitt, S., Blickhan, R., and Günther, M. Electro-Mechanical Delay in Hill-Type Muscle Models. *Journal of Mechanics in Medicine and Biology*, 12(05):1250085, 2012.

Paninski, L. Estimation of entropy and mutual information. *Neural Comput.*, 15(6):1191–1253, 2003.

Pfeifer, R., Lungarella, M., and Iida, F. Self-organization, embodiment, and biologically inspired robotics. *Science*, 318(5853):1088–1093, 2007.

Pigeon, P., Yahia, L., and Feldman, A. G. Moment arms and lengths of human upper limb muscles as functions of joint angles. *Journal of Biomechanics*, 29(10):1365–1370, 1996.

Rettig, O., Fradet, L., Kasten, P., Raiss, P., and Wolf, S. I. A new kinematic model of the upper extremity based on functional joint parameter determination for shoulder and elbow. *Gait and Posture*, 30(4):469–476, 2009.

Schreiber, T. Measuring information transfer. *Physical Review Letters*, 85(2), 2000.

Sobotta, J. *Atlas der Anatomie des Menschen*, volume 3. Elsevier Health Sciences, 2010.

Sproewitz, A., Moeckel, R., Maye, J., and Ijspeert, A. J. Learning to Move in Modular Robots using Central Pattern Generators and Online Optimization. *The International Journal of Robotics Research*, 27(3-4): 423–443, 2008. doi: 10.1177/0278364907088401.

Suissa, D. R. *Modeling, Control and Optimization in Human Motor Control: A Simulation Study of a Physiological Human Arm.* Master thesis, University of Stuttgart, 2017.

Sutton, R. S. and Barto, A. G. *Reinforcement Learning: An Introduction.* MIT Press, 1998.

Tishby, N., Pereira, F. C., and Bialek, W. The information bottleneck method. In *In Proc. of the 37-th Annual Allerton Conference on Communication, Control and Computing*, pages 368–377, 1999.

van Soest, A., Bobbert, M., Iijima, T., Shimizu, K., and Asanuma, N. The Contribution of Muscle Properise in the Control of Explosive Movments. *Biological Cybernetics*, 69(3):195–204, 1993.

von Förster, H. *Understanding Understanding - Essays on Cybernetics and Cognition.* Springer Verlag, New York, 2003.

von Uexkuell, J. A stroll through the worlds of animals and men. In Schiller, C. H., editor, *Instinctive Behavior*, pages 5–80. International Universities Press, New York, 1957. First published in 1934.

Zahedi, K. and Ay, N. Quantifying morphological computation. *Entropy*, 15(5):1887–1915, 2013.
